# Uncertainty analysis of a model of wind-blown volcanic plumes

**DOI:** 10.1007/s00445-015-0959-2

**Published:** 2015-09-07

**Authors:** Mark J. Woodhouse, Andrew J. Hogg, Jeremy C. Phillips, Jonathan C. Rougier

**Affiliations:** 1 School of Mathematics, University of Bristol, University Walk, Bristol, BS8 1TW UK; 2 School of Earth Sciences, University of Bristol, Wills Memorial Building, Queens Road, Bristol, BS8 1TW UK

**Keywords:** Uncertainty analysis, History matching, Plume model, Sensitivity analysis, Turbulent entrainment

## Abstract

Mathematical models of natural processes can be used as inversion tools to predict unobserved properties from measured quantities. Uncertainty in observations and model formulation impact on the efficacy of inverse modelling. We present a general methodology, history matching, that can be used to investigate the effect of observational and model uncertainty on inverse modelling studies. We demonstrate history matching on an integral model of volcanic plumes that is used to estimate source conditions from observations of the rise height of plumes during the eruptions of Eyjafjallajökull, Iceland, in 2010 and Grímsvötn, Iceland, in 2011. Sources of uncertainty are identified and quantified, and propagated through the integral plume model. A preliminary sensitivity analysis is performed to identify the uncertain model parameters that strongly influence model predictions. Model predictions are assessed against observations through an implausibility measure that rules out model inputs that are considered implausible given the quantified uncertainty. We demonstrate that the source mass flux at the volcano can be estimated from plume height observations, but the magmatic temperature, exit velocity and exsolved gas mass fraction cannot be accurately determined. Uncertainty in plume height observations and entrainment coefficients results in a large range of plausible values of the source mass flux. Our analysis shows that better constraints on entrainment coefficients for volcanic plumes and more precise observations of plume height are required to obtain tightly constrained estimates of the source mass flux.

## Introduction

Mathematical models provide insight into the physical processes operating in natural systems and allow investigation of the response of the system to changing conditions. Frequently, models are used as inversion tools to infer properties of a system that are difficult to measure directly from observations that can be made more easily. The characterization of uncertainty is crucial to the effective use of a model as an inversion tool. Aleatory uncertainty, that is the inevitable variations that occur in natural systems, leads to variations in observations even if conditions are seemingly identical. Epistemic uncertainty arises due to incomplete knowledge of the system, including our inability to measure precisely. Both aleatory and epistemic uncertainties impact on the inferences that can be drawn from inverse modelling; rather than achieving a single prediction of the state of the system, we instead expect a (possibly empty) set of states that are consistent with the observations.

Recent model inversion studies (e.g. Denlinger et al. 2012; Anderson and Segall 2013; Madankan et al. 2014) have adopted techniques of Bayesian inversion and calibration where observations are used to refine prior specifications of model parameters through the application of Bayes’ theorem (e.g. Kennedy and O’Hagan 2001; Craig et al. 2001). Bayesian inversion requires the specification of prior probability distributions for model inputs and the calculation of the likelihood function that quantifies the ability of the model to reproduce the observed data (Mosegaard and Tarantola 1995; Kennedy and O’Hagan 2001). The calculation of the posterior distribution is computationally demanding, particularly for a model with a large number of inputs. Thus, applications have used approximate methods in the neighborhood of the maximum likelihood estimator (e.g. Denlinger et al. 2012), Markov-chain Monte Carlo sampling (e.g. Mosegaard and Tarantola 1995; Anderson and Segall 2013), or the construction of fast surrogate models (e.g. Craig et al. 2001; Bursik et al. 2012; Madankan et al. 2014) to estimate the posterior distribution of model inputs. The uncertainty in observations can be included in the calibration, and posterior distributions provide insight into the uncertainty in model inputs.

Here, we employ an alternative method, history matching (Craig et al. 1997; Vernon et al. 2010), for examining the uncertainty in a physical model, which is related to, but differs fundamentally from, Bayesian inversion. History matching seeks to identify those model inputs that produce outputs which are consistent with uncertain observations, by ruling out model inputs that are considered implausible (Craig et al. 1997; Vernon et al. 2010), and does not involve the construction of posterior probability distributions for model inputs. The size of the model input space that is not-ruled-out by history matching is an indication of the ability of the model to predict properties of the system. Importantly, history matching allows relationships between the not-ruled-out model inputs to be examined, providing insight into how to achieve improved predictions by incorporating new observations. History matching requires sampling of the model input space and comparison of model outputs to observations, and thus requires only ‘forward’ model evaluations. It is therefore a method with general applicability. For computationally expensive models, model evaluation time may preclude an extensive sampling of the input space, and the development of statistical emulators (Santner et al. 2003; Rougier 2008; Bayarri et al. 2009; Vernon et al. 2010; Lee et al. 2011) or fast surrogate models (Bursik et al. 2012; Madankan et al. 2014) may be necessary. Our model is computationally efficient, with a single evaluation taking approximately 0.5 s on a desktop computer, allowing a thorough direct investigation of the model input space without statistical emulation.

Integral models of turbulent buoyant plumes (Morton et al. 1956) have been utilized widely to quantitatively describe the rise of plumes in industrial and environmental settings (Woods 2010), and have been extended to describe volcanic plumes (Woods 1988; Glaze and Baloga 1996; Sparks et al. 1997). Integral plume models can exploit detailed meteorological observations to describe the atmospheric stratification, moisture loading and wind (Bursik 2001; Mastin 2007; Barsotti et al. 2008; Bursik et al. 2009; Bursik et al. 2012; Degruyter and Bonadonna 2012; Woodhouse et al. 2013; Mastin 2014). The plume model can be used as a predictive tool in a ‘forward modelling’ approach, where source and atmospheric conditions are specified. Integration of the governing equations then provides predictions of plume properties such as the height of rise, and the effects of varying source and atmospheric conditions can be assessed (see, e.g. Woods 1988; Sparks et al. 1997; Glaze and Baloga 1996; Bursik 2001; Mastin 2007; Bursik et al. 2009; Degruyter and Bonadonna 2012; Woodhouse et al. 2013).

Modelling plume dynamics from specific volcanic eruptions is more difficult since, in many situations, the source conditions are not known. For hazard management applications, the estimation of source conditions is often a crucial requirement. An integral plume model can be used in an ‘inverse modelling’ approach to estimate source conditions by matching model predictions to observations by iteratively altering the model boundary conditions until a minimum in a measure of the deviation of the model output from the observation is reached. The resulting model inputs are then taken as representative of the volcanic system. Inverse modelling of this type has been referred to as ‘model calibration’ (Kennedy and O’Hagan 2001) as the output is a set of model inputs (boundary conditions and parameter values) that match model predictions to observations and can subsequently be used to predict non-observed properties. Inverse modelling using integral plume models and observations of plume heights has been used to estimate the source mass flux in reanalysis of volcanic eruptions (Bursik et al. 2012; Bonasia et al. 2012; Degruyter and Bonadonna 2012; Collini et al. 2013; Devenish 2013; Woodhouse et al. 2013; Moiseenko and Malik 2014); the unobserved source conditions are adjusted until the predicted plume height matches an observation. However, as the source conditions are usually imprecisely known, there is a large range of values over which the model inputs can be varied. Furthermore, the observed plume height can be difficult to measure accurately. For example, the studies of Bursik et al. ( 2012), Degruyter and Bonadonna ( 2012) and Woodhouse et al. ( 2013) each used a record of plume heights from the 2010 eruption of Eyjafjallajökull derived from a radar using a scanning strategy that introduces semi-discrete jumps into the measurements (Arason et al. 2011) giving measurement errors often in excess of 1 km. Mastin ( 2014) suggests that uncertainties in observational data and due to idealizations in the model formulation limit the applicability of plume models, since estimates of the source mass flux that are derived from model inversions may not be significantly better than those that can be obtained from calibrated scaling relationships (e.g. Sparks et al. 1997; Mastin et al. 2009; Degruyter and Bonadonna 2012; Woodhouse et al. 2013).

While Bayesian methods can be used to examine the uncertainty introduced by parameter calibration (e.g. Kennedy and O’Hagan 2001; Denlinger et al. 2012; Anderson and Segall 2013; Madankan et al. 2014), often inversion of volcanic plume models has varied only a single member of the set of source conditions while leaving others at fixed ‘characteristic’ values. This approach does not assess the range of possible source conditions that result in a match of the model predictions to the observation. Furthermore, the uncertainties that exist explicitly in the target observation and implicitly in the model formulation are rarely considered in inverse modelling.

In this study, we perform an uncertainty analysis of an integral plume model to examine the efficacy of plume models as inverse modelling tools that are used to estimate source conditions from observations of the plume height, using the history matching method. We take as case studies two recent eruptions in Iceland; Eyjafjallajökull in 2010 and Grímsvötn in 2011. Both of these eruptions produced plumes that ascended to high altitudes, delivering ash that was transported widely and had significant impact on aviation. This contribution is organized as follows. We first present a brief overview of the integral plume model. We then examine the sources of uncertainty in an inversion calculation using the plume model. The history-matching approach to uncertainty analysis is then introduced. We describe the methods adopted in our study, including a preliminary sensitivity analysis of model parameters, and the field observations used. We then present the results of the sensitivity analysis and history matching, interpret these in the context of volcanic ash hazard modelling and discuss the implications of our analysis for plume observations and future modelling studies.

## Overview of the integral plume model

Integral models of volcanic plumes describe changes in plume properties during the ascent of volcanic material and entrained atmospheric air (Woods 1988; Glaze and Baloga 1996; Sparks et al. 1997; Bursik 2001; Mastin 2007; Bursik et al. 2009; Bursik et al. 2012; Degruyter and Bonadonna 2012; Woodhouse et al. 2013). Typically, plume models assume a steady state, with variations in source and atmospheric conditions occurring on timescales that are much longer than the timescale for motions in the plume. For typical atmospheric conditions, this is approximately 100 s (Gill 1982).

Integral models average plume properties over a time that is long in comparison to the characteristic timescale of turbulent motion in the plume (the eddy-turnover time) (Woods 2010). The detailed transient turbulent features of the plume dynamics are therefore not described in the model. Rather, the influence of the turbulent motions on the bulk plume dynamics must be parameterized (Morton et al. 1956). The primary effect of turbulence in the plume is to engulf parcels of ambient fluid and mix these with the plume fluid (Morton et al. 1956). On viewing the plume on the long timescale of the bulk motion, this turbulent mixing appears as an inflow from the ambient into the plume which can be modelled as an ‘entrainment velocity’ at the plume margins (Morton et al. 1956; Woods 2010). Here, we denote the entrainment velocity as *U*_*e*_. Morton et al. ( 1956) demonstrated that a simple entrainment closure, with the entrainment velocity taken as linearly proportional to the plume centreline velocity, well describes laboratory scale plumes.

Atmospheric conditions can strongly influence the plume dynamics (Woods 1988; Glaze and Baloga 1996; Sparks et al. 1997). The effect of wind is particularly important as it leads to enhanced mixing of ambient air into the plume, further reducing the density contrast between the plume and the surrounding atmosphere (Bursik 2001; Woodhouse et al. 2013). Atmospheric wind has been incorporated into integral models (Bursik 2001; Barsotti et al. 2008; Bursik et al. 2009; Degruyter and Bonadonna 2012; Woodhouse et al. 2013; Devenish 2013; Mastin 2014) by adopting an entrainment model proposed by Hewett et al. ( 1971) based on laboratory studies of bent-over plumes. The entrainment velocity for a plume in a cross wind is modelled as (Hewett et al. 1971)

1 $$ U_{e} = k_{s}|U-V\cos\theta| + k_{w}|V\sin\theta|, $$

directed into the plume, where *U* is the axial centreline velocity of the plume, *V* is the horizontal speed of the wind and *𝜃* is the angle of the plume centreline to the horizontal. The entrainment model contains two entrainment coefficients, *k*_*s*_ and *k*_*w*_, that must be determined empirically. The coefficient *k*_*s*_ is the related to the entrainment that occurs due to the motion of the plume relative to the atmosphere and is equal to the entrainment coefficient of a plume in a quiescent atmosphere, while *k*_*w*_ is related to the entrainment due to the wind.

Here, we use the integral model of volcanic plumes in a wind-field developed by Woodhouse et al. ( 2013). This model can incorporate detailed atmospheric profiles from direct observations (for example, radiosonde measurements) or numerical weather prediction (NWP) models, and includes a description of the thermodynamic effects of phase changes of water in the plume. The model has four boundary conditions, representing the source mass flux *Q*_0_, exit velocity *U*_0_, source temperature *T*_0_ and the mass fraction of gas at the vent *n*_0_, and 12 parameters that specify the physical and thermodynamic properties of constituents and turbulent mixing. In addition, profiles of the atmospheric pressure, temperature, wind speed and relative humidity are required. These dimensional model inputs can be formed into 17 dimensionless groups.

## Sources of uncertainty in model inversion calculations

Model inversion in multivariable systems is non-trivial. The complicated topology of the multidimensional surface defined by the deviation of the model output from the observations (under a suitable metric; for example, the mean square deviation) can lead to many local minima or extended ‘valleys’. Finding the global minimum in this landscape is computationally challenging. Furthermore, the global minimum may differ little from a subset of the local minima, so multiple ‘acceptable’ solutions may exist. Indeed, classifying acceptable solutions requires careful consideration including an assessment of uncertainty.

Aleatory and epistemic uncertainties preclude precise agreement between model calculations and observations and so, rather than locating minima in the deviation of the model prediction from the observation, we seek model inputs such that the deviation function takes a value below a chosen threshold. Quantifying the threshold requires detailed consideration and quantification of the sources of uncertainty in the system. Following Vernon et al. ( 2010), the uncertainty in the system can be classified as (i) observational uncertainty, (ii) parameter uncertainty, (iii) simulator uncertainty, (iv) input uncertainty and (v) structural uncertainty. We discuss each of these below in the context of an inversion calculation using an integral plume model and observations of volcanic plume height made using radar.

### Observational uncertainty

Uncertainty arises in the model inversion due to errors in the measurement of natural systems. The aleatory aspect of many natural systems precludes a precise measurement. Combining multiple observations will give a distribution of measurements from which we can characterize the variability, although this risks incorporating systematic variation into our quantification of observational uncertainty.

As an example, if we consider measuring the altitude of a volcanic plume, turbulent fluctuations will give variations on the timescale that is characteristic of the turbulence, so we might take a sequence of measurements over a period of time longer than the turbulent timescale. However, there may be source variations occurring on the timescale of these measurements, leading to a systematic change in the altitude of the plume during the observations.

Observational uncertainty also arises due to lack of precision in making measurements. Direct measurements inevitably have error associated with the limited capability of instruments. Furthermore, many observations of natural systems are indirect, relying implicitly on models to relate a direct measurement to a quantity of interest. These indirect observations have additional errors due to the epistemic uncertainty in the models they adopt.

Uncertainty in the estimation of plume height by direct observation can be substantial (Tupper et al. 2009), with the records demonstrating limited accuracy in measurements (Settle 1978; Wilson et al. 1978; Sparks et al. 1997; Mastin et al. 2009) and ambiguity in the reporting of maximum plume height or neutral buoyancy height (Mastin et al. 2009). Furthermore, estimates made by ground-based observers can differ greatly for those made using satellite remote sensing technologies (Oppenheimer 1998; Tupper and Wunderman 2009).

For plume height measurements made by radar, observational errors are introduced by the scanning strategy (e.g. limited scanning angles during operational uses) (Arason et al. 2011), the finite beam width (thus the radar return signal is sourced from a range of heights around the centreline) (Arason et al. 2011), the limits of detectability for fine volcanic ash (Marzano et al. 2006; Marzano et al. 2011) and the influence of hydrometeors on the reflectivity of the plume (Rogers and Yau 1989; Guo et al. 2004; Durant et al. 2009).

Images of plumes captured in the visible spectrum (e.g. Arason et al. 2011; Bjornsson et al. 2013) or using thermal cameras (e.g. Spampinato et al. 2011; Harris 2013), from either terrestrial or satellite instruments (Oppenheimer 1998; Tupper and Wunderman 2009), can give estimates of the plume height. However, uncertainty arises due to the limited field of view, the finite resolution of images from digital cameras (Arason et al. 2011) and satellite-based instruments (Oppenheimer 1998) and when the view of the plume is obscured, e.g. by clouds. Lidar instruments can also be used to estimate the plume height, but these are typically single point for stationary instruments (e.g. Pappalardo et al. 2004) or line traverse for mobile lidar (e.g. Marenco et al. 2011) and often do not measure the maximum height.

### Parameter uncertainty

Models of natural systems introduce parameters that quantify physical properties and processes. Our knowledge of the appropriate values of these parameters is often incomplete, based on limited experimental investigations. The use of parameters fixed at ‘best’ estimated values can constrain the range of model outputs and therefore greatly influences the inferences made from model inversion.

In the integral model of volcanic plumes, there are 12 parameters. Our knowledge of appropriate values for the parameters varies greatly (Table 1). While many detailed experiments measuring thermodynamic properties of liquids and gases have been performed, few studies have examined the thermodynamics of volcanic ejecta. In plume models, thermodynamic properties of the plume constituents are often taken to be constant (with the exception of Mastin 2007 who adopts empirical relationships for the variations with temperature).

**Table 1:** Parameters in the plume model, with estimated value and associated uncertainties

Parameter (symbol)	Value	Uncertainties
Density of liquid water (*ρ*_*w*_)	999.97 kg/m ^3^	*ρ*_*w*_ is dependent on temperature (e.g. WMO 1988),
		with maximum density at 4 ^∘^C. Supercooled liquid
		water is less dense (e.g. *ρ*_*w*_=997.91 kg/*m* ^3^ at −10 ^∘^C).
		*ρ*_*w*_ also depends on the concentration of dissolved species,
		and pressure (e.g. Pruppacher and Klett 1997; Haynes 2015).
Density of solid pyroclasts (*ρ*_*s*_)	1200 kg/m ^3^	*ρ*_*s*_ varies widely by type (e.g. pumice 700–1200 kg/m ^3^;
		glass shards 2350–2450 kg/m ^3^; lithic fragments
		2700–3200 kg/m ^3^; crystal fragments 2600–5200 kg/m ^3^;
		Shipley and Sarna-Wojcicki 1982) and size
		(Taddeucci and Palladino 2002). Thus, *ρ*_*s*_ will depend on the
		composition of magma and fragmentation processes.
		Aggregation processes produce porous grains with a bulk
		density as low as 200 kg/m ^3^ (Brown et al. 2012).
Gas constant of dry air (*R*_*a*_)	287.05 J/K/kg	Well constrained.
Gas constant of water vapour (*R*_*v*_)	461.51 J/K/kg	Well constrained.
Gravitational acceleration (*g*)	9.81 m/s ^2^	Varies in the range 9.76392–9.81974 m/s ^2^ (Hirt et al. 2013)
		and varies with altitude.
Latent heat of vaporization of water at 273 K (*L*_*c*0_)	2.501×10^6^ J/kg	Well constrained (for pure water, although chemical species
		can affect the value; Pruppacher and Klett 1997).
Specific heat capacity of dry air (*C*_*a*_)	1005 J/K/kg	Varies with temperature with e.g. *C*_*a*_ = 1005 J/K/kg at
		300 K, *C*_*a*_ = 1051 J/K/kg at 600 K, *C*_*a*_ = 1142 J/K/kg
		at 1000 K (Moran and Shapiro 2006; Mastin 2007). Note,
		Woods ( 1988), Glaze and Baloga ( 1996),
		Degruyter and Bonadonna ( 2012), Woodhouse et al. ( 2013)
		use a smaller value of 998 J/K/kg; Bursik ( 2001) uses a
		value of 1000 J/K/kg.
Specific heat capacity of liquid water (*C*_*w*_)	4200 J/K/kg	Varies with temperature (and weakly with pressure,
		Haynes 2015), with *C*_*w*_ = 4219.4 J/K/kg at 273.16 K at
		1 bar (Haynes 2015), increasing to *C*_*w*_ = 4522 J/K/kg at
		243.15 K (Rogers and Yau 1989).
Specific heat capacity of solid pyroclasts (*C*_*s*_)	1100 J/K/kg	A range of values have been reported. Experimental
		measurements on air fall and basaltic scoria samples give a
		range 815–865 J/K/kg (Stroberg et al. 2010). Settle ( 1978)
		recommends a range 837–1256 J/K/kg, and modelling studies
		have typically used values at the upper end of the range (e.g.
		Wilson et al. 1978; Sparks et al. 1986; Mastin 2007;
		Neri and Macedonio 1996). Woods ( 1988) takes 1617 J/K/kg
		and many subsequent studies have adopted this.
Specific heat capacity of water vapour (*C*_*v*_)	1850 J/K/kg	Varies with temperature with e.g. *C*_*v*_=1863 J/K/kg at 300 K,
		*C*_*v*_=2014 J/K/kg at 600 K, *C*_*v*_=2288 J/K/kg at 1000 K
		(Moran and Shapiro 2006; Mastin 2007).
Entrainment coefficient in the absence of wind (*k*_*s*_)	0.09	Experiments (e.g. Morton et al. 1956; Fischer et al. 1979;
		Papanicolaou and List 1988) give a range 0.0833≤*k*_*s*_≤0.15
		(with most around 0.09). Differences are due to the measurement
		of either plume radius or fluxes (see Kaye and Linden 2004),
		source effects (Hunt and Kaye 2001), non-Boussinesq effects
		(Ricou and Spalding 1961; Rooney and Linden 1996; Woods 1997),
		and variations in entrainment with the local Richardson number for
		buoyant jets (Priestley and Ball 1955; Wang and Law 2002; Kaminski et al. 2005; Kaye 2008).
		In buoyant jets, laboratory experiments (Saffaraval et al. 2012) and numerical simulations
		(Suzuki and Koyaguchi 2010) suggest the entrainment coefficient may be as low as 0.05
		during the transition from momentum-driven to buoyancy-driven flow.
Entrainment coefficient due to wind (*k*_*w*_)	0.9	Experimental comparisons to integral model predictions of rise height (e.g.
		Hewett et al. 1971; Hoult and Weil 1972; Contini and Robins 2001) give values
		in the range 0.60≤*k*_*w*_≤0.71. Comparisons of model predictions to observations
		of chimney plumes (Hoult et al. 1969; Fay et al. 1970) give higher values,
		0.72≤*k*_*w*_≤0.9. Numerical simulations by Zhang and Ghoniem ( 1993) and
		Devenish et al. ( 2010) give values *k*_*w*_=0.7 and *k*_*w*_=0.5, respectively.

The number of significant digits in the estimated value gives an indication of the measurement accuracy. The parameters marked ‘Well constrained’ are held fixed in the parameter sensitivity screening

Laboratory experiments and high-resolution computational simulations of the fundamental governing equations can provide insight into the numerical values of the entrainment coefficients, but the values obtained for laboratory-scale plumes may not be appropriate for field-scale volcanic plumes. Morton et al. ( 1956) demonstrated that the entrainment coefficient for plumes in a quiescent environment, *k*_*s*_, calibrated in laboratory experiments well described observations of plumes over many scales, although subsequent experimental studies have not found a universal value for *k*_*s*_ (Table 1, Morton et al. 1956; Fischer et al. 1979; Papanicolaou and List 1988). In contrast, there is greater uncertainty in the entrainment coefficients for plumes rising in a cross-wind. Indeed, the values determined from experiments are smaller than those determined through model inversion applied to chimney plumes (Table 1, Hoult et al. 1969; Fay et al. 1970), perhaps since laboratory experiments do not explore the same conditions found in the natural environment. The uncertainty in entrainment coefficients for wind-blown plumes leads to substantial variation in predicted plume heights for specified source conditions, with larger values of the entrainment coefficients leading to lower plume heights due to enhanced mixing of ambient air (e.g. Morton et al. 1956; Hewett et al. 1971; Barsotti et al. 2008; Devenish et al. 2010; Degruyter and Bonadonna 2012; Woodhouse et al. 2013).

### Simulator uncertainty

Deterministic mathematical models of natural processes often involve the solution of nonlinear systems of differential equations. The analytical solutions of such systems are rarely available, so numerical methods must be adopted. We refer to the numerical solver as the simulator of the natural process. Discretization of the differential equations introduces numerical errors, known as discretization errors. Additional errors can occur in the simulator (for example, finite precision arithmetic).

For our integral model of wind-blown volcanic plumes, we have a system of coupled ordinary differential equations and algebraic equations. The numerical solution of these equations is straight-forward using standard numerical techniques. Here, we use the fourth-order Cash-Karp Runge-Kutta method with adaptive step-sizes allowing the discretization error to be controlled and providing high accuracy in the numerical solution (Press et al. 2007). The solver is computationally efficient, allowing many calculations to be performed. Therefore, in our application, we consider the simulator uncertainty to be negligible.

### Input uncertainty

Models may require additional inputs in order to represent the state of the environment at the instant the calculation is performed. These inputs have an associated uncertainty that may be difficult to quantify.

Our plume model requires a representation of the state of the atmosphere, in the form of atmospheric profiles at the vent location. We refer to these meteorological inputs as the ‘model forcing’ as the model predictions are dependent on this input. The atmospheric profiles could be obtained from direct observations and are therefore uncertain due to the observational uncertainty associated with the measurement device. For example, a radiosonde release may not take place near the volcano or at a time coincident with an eruption, and the measurements do not yield instantaneous profiles. An alternative is to use other models to generate the atmospheric profiles (e.g. NWP tools), which can be constructed at the volcanic vent and can forecast conditions at the time of an eruption. However, the model results are uncertain as they implicitly include all five sources of uncertainty.

Without detailed knowledge of the observational devices and modelling framework used to construct atmospheric profiles, it is difficult to estimate precisely the uncertainty that the forcing contributes to the plume model predictions. Here, we estimate the uncertainty in the atmospheric forcing through numerical experiments that perturb measured atmospheric profiles.

### Structural uncertainty

Models of natural processes are necessarily idealizations. In order to construct tractable mathematical descriptions, we must make simplifications of the complicated physics, adopting parameterizations of processes that we feel are important but are unable to describe fully. There may be alternative parameterizations available, and in modelling we make a choice as to which we feel is most appropriate. These approximations introduce uncertainties into the model, known as structural uncertainties (Goldstein and Rougier 2004, 2009; Vernon et al. 2010), which influence our ability to match model predictions to observations. Structural uncertainties within a model are difficult to quantify.

In many cases, parameterizations are derived from a limited number of observations in controlled laboratory settings. When adopting such a parameterization in a model of a large-scale natural process, we must appreciate that we are applying the formulation outside of the regime for which there is empirical support. For example, the wind entrainment parameterization of Hewett et al. ( 1971) is proposed on the basis of wind-tunnel experiments that adopted a uniform wind speed with altitude. In contrast, atmospheric winds typically have a vertical shear profile in the lower atmosphere, and the plumes from large volcanic eruptions may ascend beyond the tropopause and encounter jet streams with locally high wind speeds (Bursik 2001; Bursik et al. 2009).

An even greater challenge to quantifying model uncertainty is the structural uncertainty that arises due to unmodelled physical processes. In model development, we inevitably choose to neglect some physical processes as we believe they have little effect on the dominant behaviour yet would add complexity to the mathematical description. Furthermore, model development may have taken place without knowledge of some physical processes.

As an example of structural uncertainty in our wind-blown plume model, we neglect the fallout of pyroclasts. This is a pragmatic modelling decision, since it is not known how wind modifies particle settling in a bent-over plume. When the pyroclasts are fine-grained, the fallout of solids has little effect on the plume dynamics, since the particles rapidly transfer their heat to the gaseous phases and the mass lost from the plume due to fallout is only a small proportion of the total mass of the plume (Woods and Bursik 1991; Sparks et al. 1997). However, if the eruption produces coarse-grained material, the heat transfer occurs on a longer timescale so that fallout may occur before thermal equilibrium is reached (Woods and Bursik 1991; Sparks et al. 1997). Fallout of coarse grains can then have a significant effect on the plume dynamics. Therefore, the structural uncertainty introduced by our neglect of particle fallout could be significant for eruptions producing large pyroclasts, but is likely small for fine-grained eruption columns.

Additionally, our model assumes that the pressure in the plume is equal to the atmospheric pressure throughout the ascent. This assumption may not be appropriate very near to the vent where the erupted material can have a substantial overpressure (Woods and Bower 1995; Ogden et al. 2008b; Saffaraval et al. 2012). Large overpressure alters the flow dynamics (Woods and Bower 1995; Ogden et al. 2008b; Ogden et al. 2008a), in particular the turbulent mixing processes, such that a different parameterization of entrainment is required (Saffaraval et al. 2012). However, the pressure in the near vent jet rapidly adjusts to atmospheric pressure (Saffaraval et al. 2012) and therefore, while the neglect of physical processes induced by the pressure disequilibrium introduces structural uncertainty in the model, we expect the model results to be little affected by the simplified description (Saffaraval et al. 2012).

## History matching

In general, we can view our simulator as a function, ***f***, mapping a set of inputs ***x*** onto a set of model outputs ***y*** ^*mod*^ using specified parameters ***𝜃***,

2 $$ \boldsymbol{y}^{mod} = \boldsymbol{f}(\boldsymbol{x};\boldsymbol{\theta}). $$

Here, ***x*** denotes the ‘active’ inputs that we seek to determine from model inversion, and ***𝜃*** denotes model parameters that remain fixed. We assume that the set of all possible inputs, $\mathcal {X}$, is non-empty (i.e. there is at least one input that produces a model output) so that it is possible to run the simulator. In practice, the model is only useful if many inputs covering the range of natural phenomena can be used in the simulator, so the set $\mathcal {X}$ is typically large. In addition, we assume the model is deterministic, so that ***f***(***x***;***𝜃***) produces the same output ***y*** ^*mod*^ each time a simulation is performed. However, the model output could be the same for two different inputs.

In model inversion, we seek to find the set of inputs $\boldsymbol {x}\in \mathcal {X^{\ast }}\subset \mathcal {X}$ that give rise to plausible model output with respect to the physical observables ***y*** ^*phys*^ accounting for uncertainty. We will do this through a *history matching* approach whereby we attempt to determine all elements of $\mathcal {X^{\ast }}$, rather than attempt to find the (probability density function for the) best match as is done in model calibration (Kennedy and O’Hagan 2001; Rougier 2007; Denlinger et al. 2012; Madankan et al. 2014).

Observational uncertainty means that the values of the quantities we observe differ from the actual physical values, i.e. ***y*** ^*obs*^=***y*** ^*phys*^+***𝜖*** ^*obs*^, where ***y*** ^*obs*^ is the observed quantity corresponding to ***y*** ^*phys*^ and ***𝜖*** ^*obs*^ is the observational error, which we assume is uncorrelated with ***y*** ^*phys*^ (Craig et al. 1997; Vernon et al. 2010). Therefore, while we wish to match ***y*** ^*mod*^ to ***y*** ^*phys*^, we must instead be satisfied with a match to ***y*** ^*obs*^, but we should take account of ***𝜖*** ^*obs*^.

Parameter uncertainty, simulator uncertainty, input uncertainty and structural uncertainty can be combined into a model discrepancy ***𝜖*** ^*md*^ (Vernon et al. 2010), such that, even if the physical inputs (denoted by ***x*** ^*phys*^) that give rise to the observables ***y*** ^*phys*^ were known, then ***y*** ^*phys*^=***f***(***x*** ^*phys*^;***𝜃***)+***𝜖*** ^*md*^. The model discrepancy quantifies the ability of the simulator to reproduce the physical state of the system being modelled when the physical inputs are used, and is estimated by examination of model outputs using a variety of model inputs.

Following Vernon et al. ( 2010), we introduce *implausibility measures*, *I*_*i*_(***x***) for each of the model outputs which are labelled with the index *i*, to quantify the discrepancy in the model predictions $y_{i}^{mod}$ of $y_{i}^{phys}$ while accounting for uncertainty, defined for a general system in which probability density functions are specified for the model discrepancy (e.g. for a model that includes stochastic forcing) and observational error as

3 $$ {I^{2}_{i}}(\boldsymbol{x}) = \frac{\left( y_{i}^{obs}-f_{i}\left( \boldsymbol{x};\boldsymbol{\theta}\right)\right)^{2}}{\text{Var} \left( \epsilon_{i}^{md}\right)+\text{Var}\left( \epsilon_{i}^{obs}\right)}. $$

Therefore, the implausibility measure ${I_{i}^{2}}$ is the squared deviation of the model prediction from the observation scaled by the uncertainty in the model and observation. Separate implausibility measures are constructed for each model output–observation pair and can be combined to give an overall indication of the discrepancy (Vernon et al. 2010), but here we focus on a single model output and therefore adopt a single implausibility measure, *I*(***x***), (additional model outputs can then be assessed sequentially).

The implausibility measure is large when the model output differs from the observation by an amount that is unlikely to be due only to model and observational uncertainty. The model inputs giving rise to a large implausibility measure are then considered as unlikely to be appropriate representations of the physical state of the system, and these regions of the model input space can be visualized by projecting the implausibility measure into subspaces of the model inputs. Visualization of the implausibility measure provides insight into the effect of uncertainty on model predictions. In order to rule-out regions of the model input space as implausible, an implausibility cut-off *I*_*p*_ must be defined. The plausible input space $\mathcal {X}^{\ast }$ is then populated with inputs such that $I\left (\boldsymbol {x}\right ) < I_{p}$. Vernon et al. ( 2010) recommends *I*_*p*_=3 based on the 3*σ* rule (implying, in some generality, *I*(***x*** ^*phys*^)<3 with probability greater than 0.95, Pukelsheim 1994), and we adopt this value here.

In this study, the variances of the model discrepancy and observational uncertainty are specified to be constants, and we consider a single model output. For this simple situation, it is straight-forward to assess changes to the implausibility measure if the observational uncertainty or model discrepancy is reduced. For example, if we suppose that our measurement of *y* ^*obs*^ improves such that the variance in the observational uncertainty is reduced to *α*Var(*𝜖* ^*obs*^) (which can be achieved by scaling the distribution of the observational uncertainty by $\sqrt {\alpha }\epsilon ^{obs}$) and the variance of the model discrepancy is reduced to *β*Var(*𝜖* ^*md*^) for dimensionless factors 0<*α*≤1 and 0<*β*≤1, then we can define a new implausibility measure, *I* ^(*new*)^, as

4 $$ I^{(new)}=\sqrt{\frac{\left( y^{obs}-f\left( \boldsymbol{x};\boldsymbol{\theta}\right)\right)^{2}}{\alpha\text{Var}\left( \epsilon^{obs}\right) + \beta\text{Var}\left( \epsilon^{md}\right)}}.  $$

**Fig. 1:**
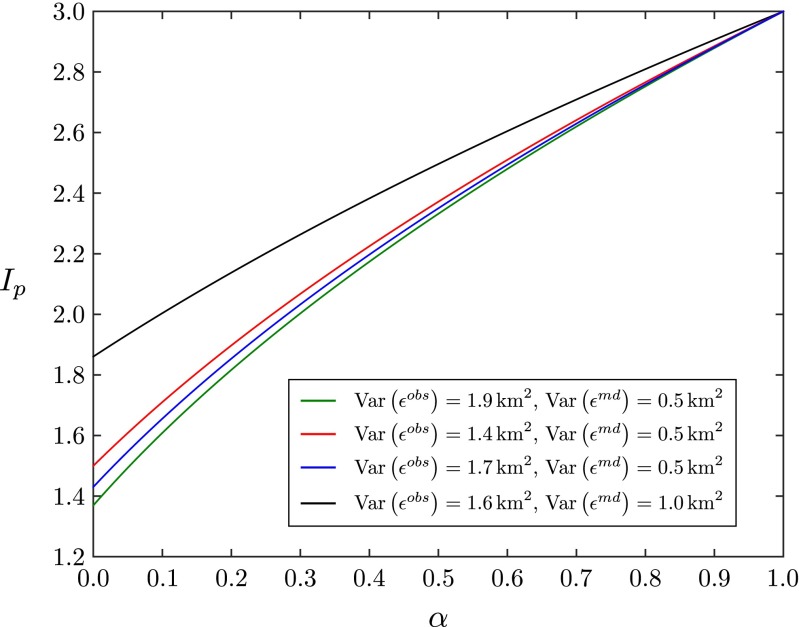
Threshold for the implausibility measure, *I*_*p*_, as a function of the factor by which the observational error is reduced, *α*, while the model discrepancy is not changed (*β*=1). If the observational error $\text {Var}\left (\epsilon ^{obs}\right )$ is reduced to $\alpha \text {Var}\left (\epsilon ^{obs}\right )$ with 0<*α*≤1, then the threshold on the implausibility measure *I*_*p*_ below which the model output can be considered a plausible match to the observation is decreased. Four examples are shown, with $\text {Var}\left (\epsilon ^{obs}\right )=1.9~\text {km}^{2}$, $\text {Var}\left (\epsilon ^{md}\right )=0.5~\text {km}^{2}$ (*green line*), $\text {Var}\left (\epsilon ^{obs}\right )=1.4~\text {km}^{2}$, $\text {Var}\left (\epsilon ^{md}\right )=0.5~\text {km}^{2}$ (*red line*), $\text {Var}\left (\epsilon ^{obs}\right )=1.7~\text {km}^{2}$, $\text {Var}\left (\epsilon ^{md}\right )=0.5~\text {km}^{2}$ (*blue line*) and $\text {Var}\left (\epsilon ^{obs}\right )=1.6~\text {km}^{2}$, $\text {Var}\left (\epsilon ^{md}\right )=1.0~\text {km}^{2}$ (*black line*)

The new implausibility measure can be related to the original measure, *I*, through,

5 $$ \frac{I^{(new)}}{I} = \sqrt{\frac{\text{Var}\left( \epsilon^{obs}\right) + \text{Var}\left( \epsilon^{md}\right)}{\alpha\text{Var}\left( \epsilon^{obs}\right) + \beta\text{Var}\left( \epsilon^{md}\right)}},  $$

and therefore, taking *I* ^(*new*)^=3 as a threshold on the plausible model output, a corresponding threshold on the original implausibility measure can be calculated (Fig. 1). Through Eq. 5, the history-matching results can be updated if new observations allow the observational uncertainty or the uncertainty due to the model forcing to be reduced, simply by calculating the appropriate threshold on the original implausibility measure, provided the target observations and model inputs are unchanged.

## Methods

### Parameter sensitivity screening

History matching requires a thorough investigation of the model input space. The number of model evaluations grows exponentially with the dimension of the input space, so that, when the input space is large, the number of calculations required to adequately cover the input space may be prohibitive. We therefore attempt to reduce the dimension of the input space wherever possible. The approach that we follow here is to assess the sensitivity of the model to the parameters using typical conditions. Only those parameters with a substantial influence on the variation of the model output are included as an active input, while those that have little effect on the model solutions are held fixed. This involves a subjective decision on the threshold of sensitivity that determines which parameters are included in the active input set, a decision that is guided by analysis of the model outputs. We note that this preliminary parameter screening is not necessary for the subsequent history matching which could be performed with all parameters allowed to vary. However, the reduction in the size of the input space achieved by removing the non-influential parameters allows for a more detailed examination of the remaining model inputs without greatly affecting the variability in model predictions that is due to uncertain parameters.

Despite the very rapid numerical integration of the governing equations, an investigation of the 17 dimensional model input space is prohibitively time consuming. We therefore perform an initial sensitivity analysis of the nine most poorly constrained model parameters (see Table 1), using the variance-based sensitivity analysis of Saltelli et al. ( 2010). The sensitivity of the model outputs to changes in parameters are quantified using sensitivity indices; here, we compute the first-order and total-effect sensitivity indices. Full details of the sensitivity indices are given in Saltelli et al. ( 2010), and a similar methodology has been employed by Scollo et al. ( 2008).

The calculation of the sensitivity indices requires the evaluation of multidimensional integrals over the model parameter space, and is therefore computationally expensive for a model with a large number of parameters. Saltelli et al. ( 2010) gives estimators of the integrals required to compute the sensitivity indices that can be obtained from a random, space-filling sampling of the parameter space. Here, we use a Latin hypercube design with a maximin criteria, iteratively adjusting the placement of sampling points in the Latin hypercube to maximize the minimum distance between points (Morris and Mitchell 1995). Confidence intervals on the first-order and total-effect sensitivity indices are estimated by a bootstrap of the Latin hypercube samples (Archer et al. 1997; Yang 2011).

For a sensitivity analysis focused on the model parameters, we hold the other model inputs (i.e. the model boundary conditions and the meteorological forcing) fixed. Therefore, the sensitivity analysis is strictly only informative on the sensitivity of the model predictions to changes in parameters for the particular choice of boundary conditions and atmospheric conditions. We therefore select atmospheric conditions that are representative of expected conditions during the Eyjafjallajökull and Grímsvötn eruptions. In particular, we choose to use atmospheric data obtained from radiosonde ascent at Keflavík International Airport at 1200 (all times given are UTC) on 11 May 2010, and take vent radius *L*_0_=80 m, exit velocity *U*_0_ = 80 m/s, source temperature *T*_0_ = 1000 K and gas mass fraction at the vent *n*_0_ = 0.03.

### History matching

Following the sensitivity screening of the model parameters, those parameters that have a substantial influence on the model output are included in the set of active inputs alongside the unknown boundary conditions, and the uncertainty analysis on the model input set is performed following the history-matching approach. In this study, we use observations of the height of volcanic plumes derived from radar instruments during the eruptions of Eyjafjallajökull in 2010 and Grímsvötn in 2011.

**Fig. 2:**
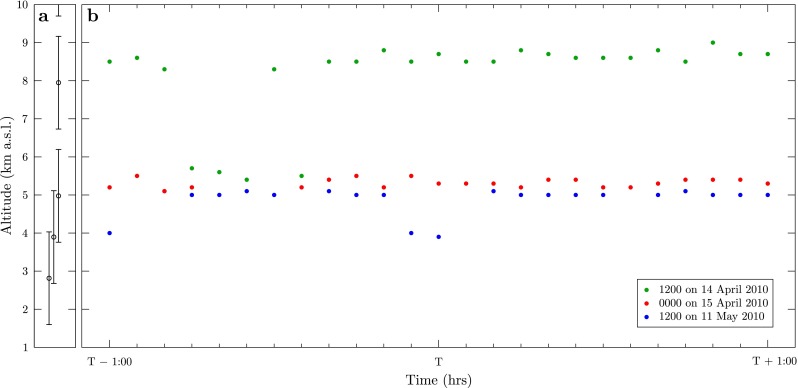
Radar-derived plume top heights at Eyjafjallajökull. **a** The radar scanning levels adopted throughout the 2010 eruption (*circles*) and beam widths at Eyjafjallajökull (*bars*). The finite beam width and steps in scanning heights result in observational uncertainties in the radar-derived plume heights. **b** Plume top heights at Eyjafjallajökull derived from the C-band radar at Keflavík on 14 April 2010 (*green points*, *T* = 1200), 15 April 2010 (*red points*, *T* = 0000) and 11 May 2010 (blue points, *T* = 1200)

**Table 2:** Plume height target values and associated quantified uncertainties for history matching

Event location and time	Plume height target value	Observational uncertainty, $\text {Var}\left (\epsilon ^{obs}\right )$	Model discrepancy, $\text {Var}\left (\epsilon ^{md}\right )$
Eyjafjallajökull 14 April 2010 at 1200	8.6 km	1.9 km ^2^	0.5 km ^2^
Eyjafjallajökull 15 April 2010 at 0000	5.3 km	1.4 km ^2^	0.5 km ^2^
Eyjafjallajökull 11 May 2010 at 1200	5.0 km	1.7 km ^2^	0.5 km ^2^
Grímsvötn 22 May 2011 at 0500	19.2 km	1.6 km ^2^	1.0 km ^2^

During the Eyjafjallajökull eruption in April and May 2010, a fixed C-band weather radar located at Keflavík International Airport, 155 km west of the volcano, made reflectivity scans every 5 min giving a high temporal resolution data set of the plume height (Arason et al. 2011). However, the distance of the volcano from the radar, the scanning strategy employed and the limitations of the C-band radar in detecting fine ash and distinguishing hydrometeors, result in large observational uncertainty in the inferred plume height of up to 2.5 km (Fig. 2 and Arason et al. 2011).

**Fig. 3:**
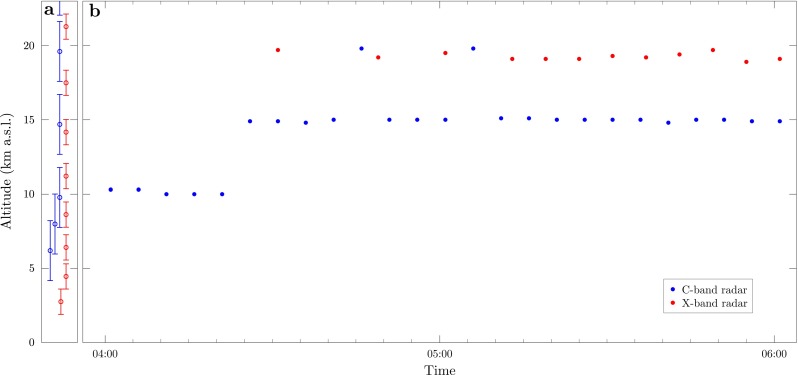
Radar-derived plume top heights at Grímsvötn. **a** The scanning levels (*circles*) and beam widths at Grímsvötn (*bars*) of the C-band (*blue*) and X-band (*red*) radars are shown. The finite beam width and steps in scanning heights result in observational uncertainties in the radar-derived plume heights. **b** Plume top heights at Grímsvötn from 0400 to 0600 on 22 May 2011 derived from the C-band (*blue points*) and X-band (*red points*) radars

We consider three times during the Eyjafjallajökull eruption: 1200 on 14 April 2010 and 0000 on 15 April 2010 during the first explosive phase of the eruption (14–18 April 2010), and 1200 on 11 May 2010 during the second explosive phase (5–17 May 2010). The first explosive phase of the Eyjafjallajökull eruption was characterized by phreatomagmatic activity due to interaction of magma with glacial ice on the summit, and the production of fine ash (Gudmundsson et al. 2012). The plume at 1200 on 14 April 2010 reached a higher altitude than the plume at 0000 on 15 April. Variations in the plume height during the first explosive phase have been attributed to changes in the wind strength with a constant source mass flux using simple inversion from radar-derived plume heights (Woodhouse et al. 2013). The second explosive phase was less vigorous than the first explosive phase (Gudmundsson et al. 2012). Figure 2 shows the variation in the plume height as measured by the C-band radar at Keflavík, and Table 2 gives the target plume height (here taken as the median plume height in the 2-h period centred on the observation time) and associated observational uncertainty.

The Grímsvötn eruption in May 2011 was monitored by two radar instruments; the fixed C-band radar at Keflavík International Airport and a mobile X-band radar that was deployed near to the volcano during the eruption (Petersen et al. 2012). The C-band radar at Keflavík was 257 km from Grímsvötn and produced plume height measurements every 5 min during the eruption (Petersen et al. 2012). The X-band radar was located 75 km from Grímsvötn and was operational from 0327 on 22 May 2011, approximately 8.5 h after the start of the eruption (Petersen et al. 2012). Operational challenges of the field deployment led to a fragmented record of plume height (Petersen et al. 2012), and the scanning strategy employed results in only a slight improvement in the vertical resolution of the plume height in comparison to the fixed C-band radar (Fig. 3 and Petersen et al. 2012). The X-band radar gives at plume height of 19.2 km at 0500 on 22 May 2011, and we estimate an uncertainty of 1.6 km in this measurement (Table 2).

**Table 3:** Range of values for active model inputs in history matching

Parameter (symbol)	Range
Source mass flux (*Q*_0_)	10^3^– 10^9^ kg/s
Exit velocity (*U*_0_)	10– 500 m/s
Source temperature (*T*_0_)	800– 1500 K
Gas mass fraction at source (*n*_0_)	0.001–0.25
Specific heat capacity of solid pyroclasts (*C*_*s*_)	815– 1617 J/K/kg
Specific heat capacity of dry air (*C*_*a*_)	998– 1142 J/K/kg
Entrainment coefficient in the absence of wind (*k*_*s*_)	0.07–0.16
Entrainment coefficient due to wind (*k*_*w*_)	0.1–1.2

**Fig. 4:**
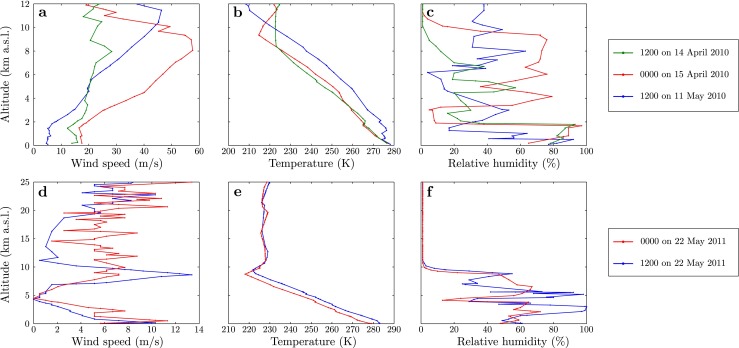
Meteorological profiles as measured by radiosonde ascent at Keflavík International Airport. Profiles of **a**, **d** wind speed, **b**, **e** temperature and **c**, **f** relative humidity as a function of altitude are shown. The data in **a**–**c** are used to describe the atmospheric conditions at Eyjafjallajökull on 14 April 2010 at 1200 (green), 15 April 2010 at 0000 (*red*) and 11 May 2010 at 1200 (*blue*). In **d**–**f**, radiosonde data obtained on 22 May 2011 at 0000 (*blue*) and 1200 (*red*) are shown, which are used to describe the atmospheric conditions at Grímsvötn. Note the noisy signal at low wind speed (**d**) and for the relative humidity (**c**, **f**)

The atmospheric conditions during the Eyjafjallajökull and Grímsvötn eruptions are not known precisely. No direct measurements of atmospheric properties are available at the location of the volcano. Measurements of atmospheric profiles are made twice daily at Keflavík International Airport by radiosondes. These are the only direct measurements available during the eruptions, and we use these measured atmospheric properties. The adoption of radiosonde measurements to describe the atmospheric structure at the volcanoes therefore introduces uncertainties into the model solutions which are quantified in the model discrepancy. Meteorological conditions measured by radiosonde are shown in Fig. 4. For the Eyjafjallajökull examples, we estimate the model discrepancy to be Var(*𝜖* ^*md*^)=500 m^2^ due primarily to uncertainty in the atmospheric observations. The Grímsvötn observation at 0500 on 22 May 2011 has no coincident atmospheric observation, lying between the radiosonde releases at 0000 and 1200 that show differences in conditions (Fig. 4d–f). This leads to increased uncertainty in the model which we estimate by comparing the model predictions obtained using atmospheric soundings taken at 0000 and 1200 on 22 May 2010, with fixed source conditions. We find the different atmospheric conditions lead to model plume heights that differ by approximately 1 km and adopt this value as an estimate of the uncertainty due to imprecise meteorological inputs (Table 2).

In this initial analysis, we take large ranges for the active model inputs (Table 3) to avoid excluding values that might give acceptable model predictions even though experts may judge these values to be unrepresentative of the physical system. Indeed, our analysis examines the ability of the model to reproduce observations and therefore it is of interest to determine the extent to which observations constrain model inputs to ranges that are likely representative of the volcanic setting. We make no further probability judgements on the values of each of the active inputs. Further analysis following the preliminary history matching could employ prior probability distributions, perhaps guided by expert judgments. The range of values taken for the active inputs in our history matching analysis is given in Table 3. For the model parameters, the range spans the values used in previous studies (see Table 1), whereas the model boundary conditions take values that describe a variety of source conditions. To sample the space of active model inputs, we construct a space filling Latin hypercube with a maximin design (Morris and Mitchell 1995).

## Results

### Parameter sensitivity screening

The sensitivity indices for the poorly constrained model parameters are shown in Table 4. The slow convergence of the estimators to the multidimensional integrals required for computation of the sensitivity indices means a large number of sample points in the Latin hypercube are needed. Here, we have taken 1 million sample points, resulting in 11 million forward model runs. Such large sample sizes are possible here as the model calculations are rapid.

**Table 4:** Sensitivity indicesfor plume model parameters

Parameter (symbol)	First-order sensitivity index	95 % Confidence interval from bootstrap
Density of liquid water (*ρ*_*w*_)	2.4×10^−9^	[−4.3×10^−9^,6.4×10^−9^]
Density of solid pyroclasts (*ρ*_*s*_)	5.6×10^−5^	[−1.3×10^−5^,2.0×10^−4^]
Gravitational acceleration (*g*)	4.2×10^−4^	[−4.2×10^−4^,3.5×10^−4^]
Specific heat capacity of dry air (*C*_*a*_)	7.7×10^−2^	[6.8×10^−2^,8.0×10^−2^]
Specific heat capacity of liquid water (*C*_*w*_)	2.5×10^−8^	[−3.1×10^−5^,6.6×10^−5^]
Specific heat capacity of solid pyroclasts (*C*_*s*_)	5.3×10^−1^	[5.2×10^−1^,5.5×10^−1^]
Specific heat capacity of water vapour (*C*_*v*_)	1.8×10^−4^	[−3.0×10^−4^,3.8×10^−4^]
Entrainment coefficient in absence of wind (*k*_*s*_)	2.6×10^−2^	[2.2×10^−2^,2.9×10^−2^]
Entrainment coefficient due to wind (*k*_*w*_)	3.2×10^−1^	[3.2×10^−1^,3.4×10^−1^]
	Total effects sensitivity index	95 % Confidence interval from bootstrap
Density of liquid water (*ρ*_*w*_)	7.6×10^−14^	[5.0×10^−14^,1.0×10^−13^]
Density of solid pyroclasts (*ρ*_*s*_)	3.1×10^−5^	[3.1×10^−5^,3.1×10^−5^]
Gravitational acceleration (*g*)	4.2×10^−4^	[4.2×10^−4^,4.3×10^−4^]
Specific heat capacity of dry air (*C*_*a*_)	9.6×10^−2^	[9.6×10^−2^,9.6×10^−2^]
Specific heat capacity of liquid water (*C*_*w*_)	6.7×10^−6^	[6.7×10^−6^,6.8×10^−6^]
Specific heat capacity of solid pyroclasts (*C*_*s*_)	5.7×10^−1^	[5.7×10^−1^,5.8×10^−1^]
Specific heat capacity of water vapour (*C*_*v*_)	3.4×10^−4^	[3.4×10^−4^,3.4×10^−4^]
Entrainment coefficient in absence of wind (*k*_*s*_)	3.3×10^−2^	[3.2×10^−2^,3.3×10^−2^]
Entrainment coefficient due to wind (*k*_*w*_)	3.7×10^−1^	[3.7×10^−1^,3.7×10^−1^]

A ranking of the importance of the model parameters can be determined from the sensitivity analysis. Here, we find that the solids heat capacity *C*_*s*_, wind entrainment coefficient *k*_*w*_, heat capacity of dry air *C*_*a*_ and no-wind entrainment coefficient *k*_*s*_ (in descending order) have largest effect on the variation in model outputs. The sensitivity indices of the other model parameters are orders of magnitude smaller. In other meteorological conditions, the ordering of the influential parameters (*C*_*s*_, *k*_*w*_, *C*_*a*_ and *k*_*s*_) changes, but no other parameters enter this set. In particular, if the wind speed is reduced then the sensitivity indices for the parameter *k*_*s*_ increase by an order of magnitude (results not given here). Given the sensitivity of the model to these parameters and the uncertainty in appropriate values, we include the parameters *C*_*a*_, *C*_*s*_, *k*_*s*_ and *k*_*w*_ in the set of active model inputs.

### History matching

The visualization of the multidimensional implausibility function is challenging. Here, we use projections of the minimum of the implausibility measure onto two-dimensional sections. Thus, we compute the smallest implausibility of all points in our design which have *a*≤*x*_*i*_<*b* and *c*≤*x*_*j*_<*d* for appropriate choices of *a*, *b*, *c* and *d*. The minimum projection metric ensures that, for a model input *x*_*i*_ to be considered implausible, the implausibility measure exceeds the implausibility threshold for all possible values of *x*_*j*_ for *j*≠*i*.

**Fig. 5:**
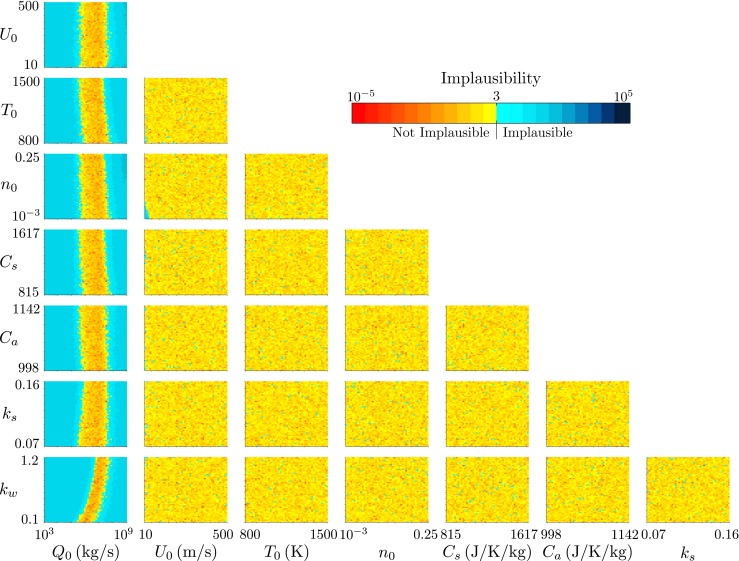
The projection of the minimum implausibility measure onto two-variable planes for history matching to the radar-derived plume height at Eyjafjallajökull at 1200 on 14 April 2010

**Fig. 6:**
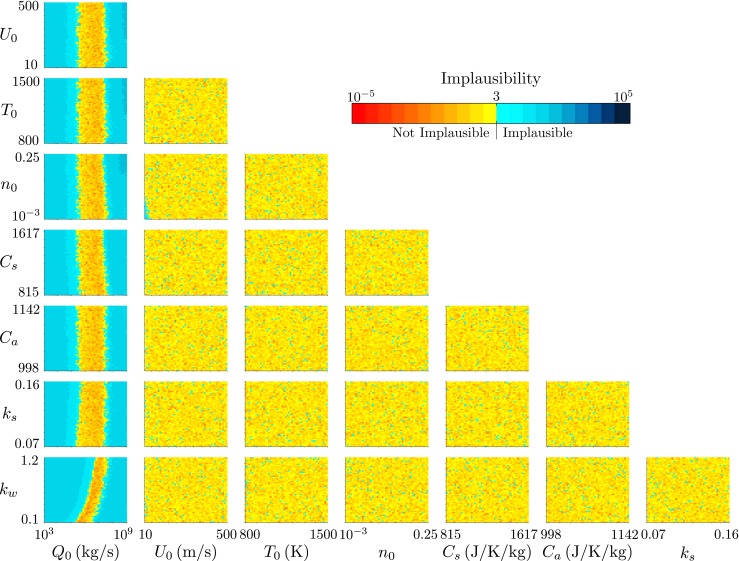
The projection of the minimum implausibility measure onto two-variable planes for history matching to the radar-derived plume height at Eyjafjallajökull at 0000 on 15 April 2010

We consider first the observations of plume height from Eyjafjallajökull at 1200 on 14 April 2010 and 0000 on 15 April 2010. The history matching results for 1200 on 14 April as visualized by projections of the minimum of the implausibility measure onto all combinations of two-variable planes are shown in Fig. 5. Several immediate inferences can be made. Firstly, there is a limited range of the source mass flux *Q*_0_ that can be input into the model such that a plume height prediction that plausibly matches the observed height is obtained; we can refer to these values as plausible predictions of the source mass flux and denote the values as $Q_{0}^{\ast }$. On 14 April 2010, we find $2.6\times 10^{5} \leq Q_{0}^{\ast } \leq 7.5\times 10^{7}\,\mathrm {kg/s}$, whereas we obtain $2.1\times 10^{5} \leq Q_{0}^{\ast } \leq 4.6\times 10^{7}\,\mathrm {kg/s}$ on 15 April 2010. The plausible values of the source mass flux $Q_{0}^{\ast }$ are strongly dependent on the choice of the wind entrainment coefficient *k*_*w*_, with higher values of $Q_{0}^{\ast }$ when *k*_*w*_ is large. The other model inputs have weaker influence on the plausible values of the source mass flux. For example, there is evidence that values of $Q_{0}^{\ast }$ increase with decreasing source temperature *T*_0_, and there is a slightly smaller range of plausible source mass flux when *k*_*s*_ is large. The plume height observation is insufficient to reduce the range of the input variables other than the source mass flux, with the exception of a region with both *U*_0_ and *n*_0_ small that is implausible. Similar results are obtained from the history matching for 0000 on 15 April (Fig. 6).

The projection of the implausibility measure onto two-variable planes hides the higher-order dependencies of the model predictions on the model inputs. Further insight can be gained from an examination of the implausibility measure in cuts through the input space. In particular, we choose small intervals of the source mass flux, spanning the range of not-implausible source mass flux. Within each interval, we examine the implausibility measure as functions of *k*_*w*_ and the remaining input variables.

**Fig. 7:**
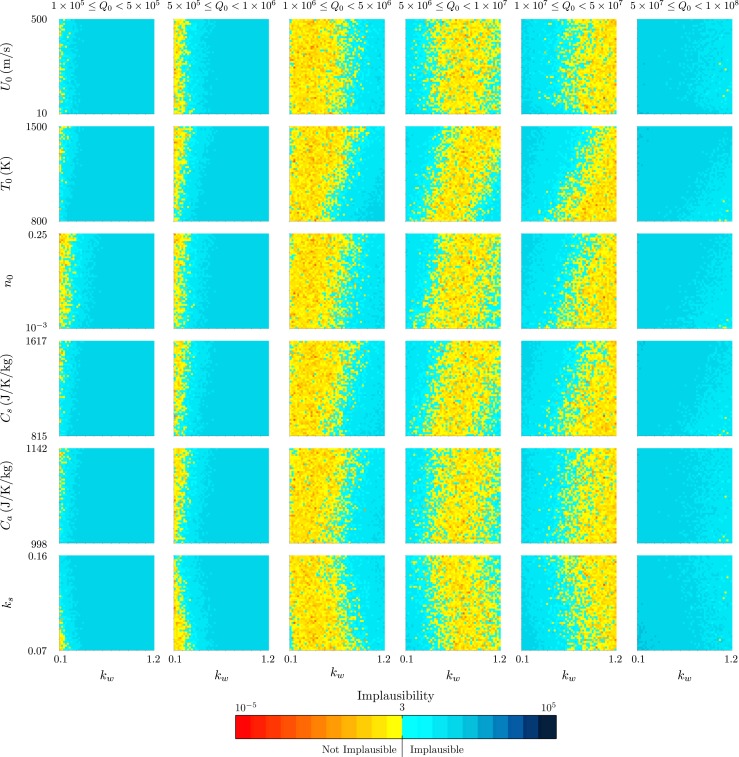
The projection of the minimum implausibility measure onto two-variable planes within intervals of the source mass flux *Q*_0_ for history matching to the radar-derived plume height at Eyjafjallajökull at 1200 on 14 April 2010

**Fig. 8:**
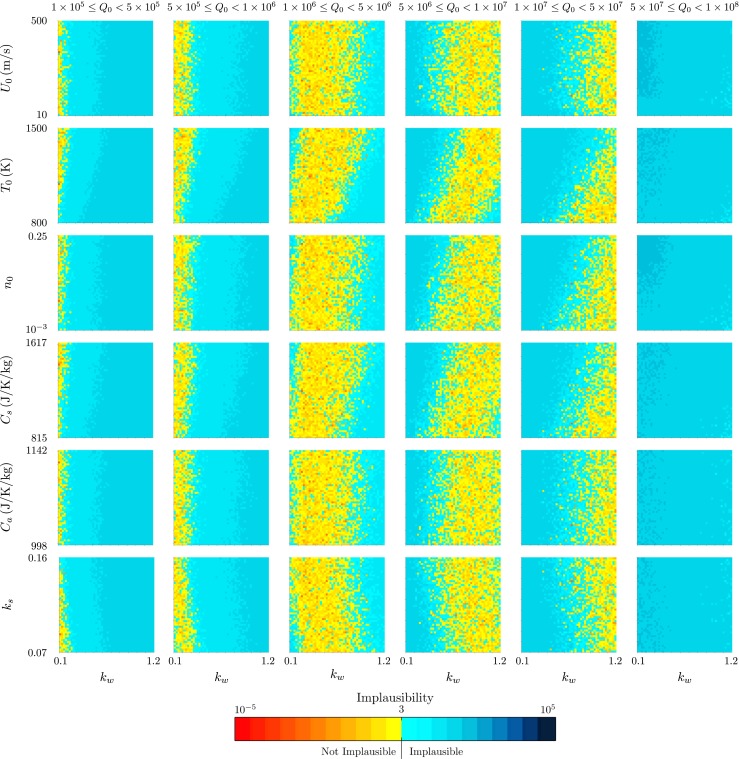
The projection of the minimum implausibility measure onto two-variable planes within intervals of the source mass flux *Q*_0_ for history matching to the radar-derived plume height at Eyjafjallajökull at 0000 on 15 April 2010

Figures 7 and 8 demonstrate the additional relationships between model inputs that can be drawn out by this visualisation. The strong dependence of the source mass flux *Q*_0_ on the value of the wind entrainment coefficient *k*_*w*_ is again apparent. Additionally, we see a three-way interaction between the source mass flux *Q*_0_, source temperature *T*_0_ and the wind entrainment coefficient *k*_*w*_, with higher values of *T*_0_ ruled-out unless *k*_*w*_ is sufficiently high (see the *T*_0_ row and columns 3–5 in Figs. 7 and 8). Similar relationships are seen in the gas mass fraction *n*_0_ and solids heat capacity *C*_*s*_ inputs. In contrast, the no-wind entrainment coefficient *k*_*s*_ is (weakly) inversely related to the wind entrainment coefficient *k*_*w*_; the not-ruled out values of *k*_*s*_ decrease as *k*_*w*_ increases.

**Fig. 9:**
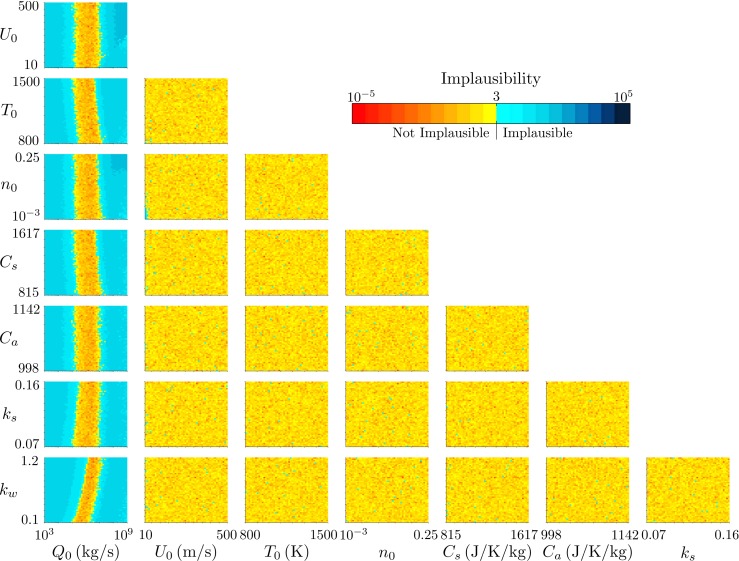
The projection of the minimum implausibility measure onto two-variable planes for history matching to the radar-derived plume height at Eyjafjallajökull at 1200 on 11 May 2010

**Fig. 10:**
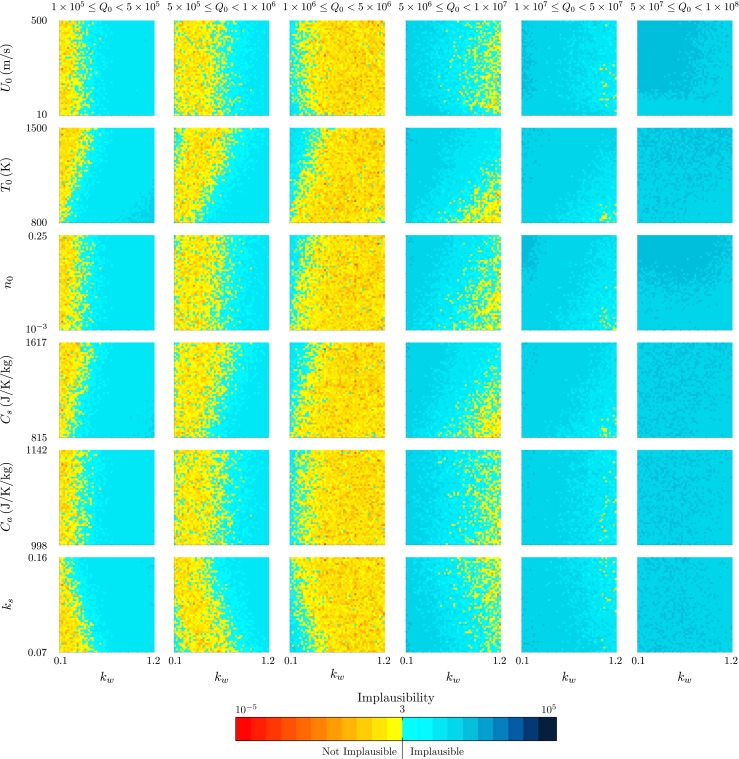
The projection of the minimum implausibility measure onto two-variable planes within intervals of the source mass flux *Q*_0_ for history matching to the radar-derived plume height at Eyjafjallajökull at 1200 on 11 May 2010

We consider next the plume from Eyjafjallajökull at 1200 on 11 May 2010, during the second explosive phase of the eruption. Figure 9 shows projections of the implausibility measure onto two-variable planes, and Fig. 10 demonstrates the relationship between the model inputs and the wind entrainment coefficient *k*_*w*_ within intervals of the source mass flux *Q*_0_. Comparison of Fig. 9 with Figs. 5 and 6 shows that the range of not-implausible source mass flux on 11 May 2010 ($1.0\times 10^{5} \leq Q_{0}^{\ast } \leq 2.1\times 10^{7}\,\mathrm {kg/s}$) is at lower values than for 14 and 15 April 2010. A stronger inverse relationship between the entrainment coefficients is seen in Fig. 10 than in Figs. 7 and 8. In other respects, the dependencies of the implausibility measure on the input parameters for 1200 on 11 May 2010 are similar to those seen for 14 and 15 April 2010.

**Fig. 11:**
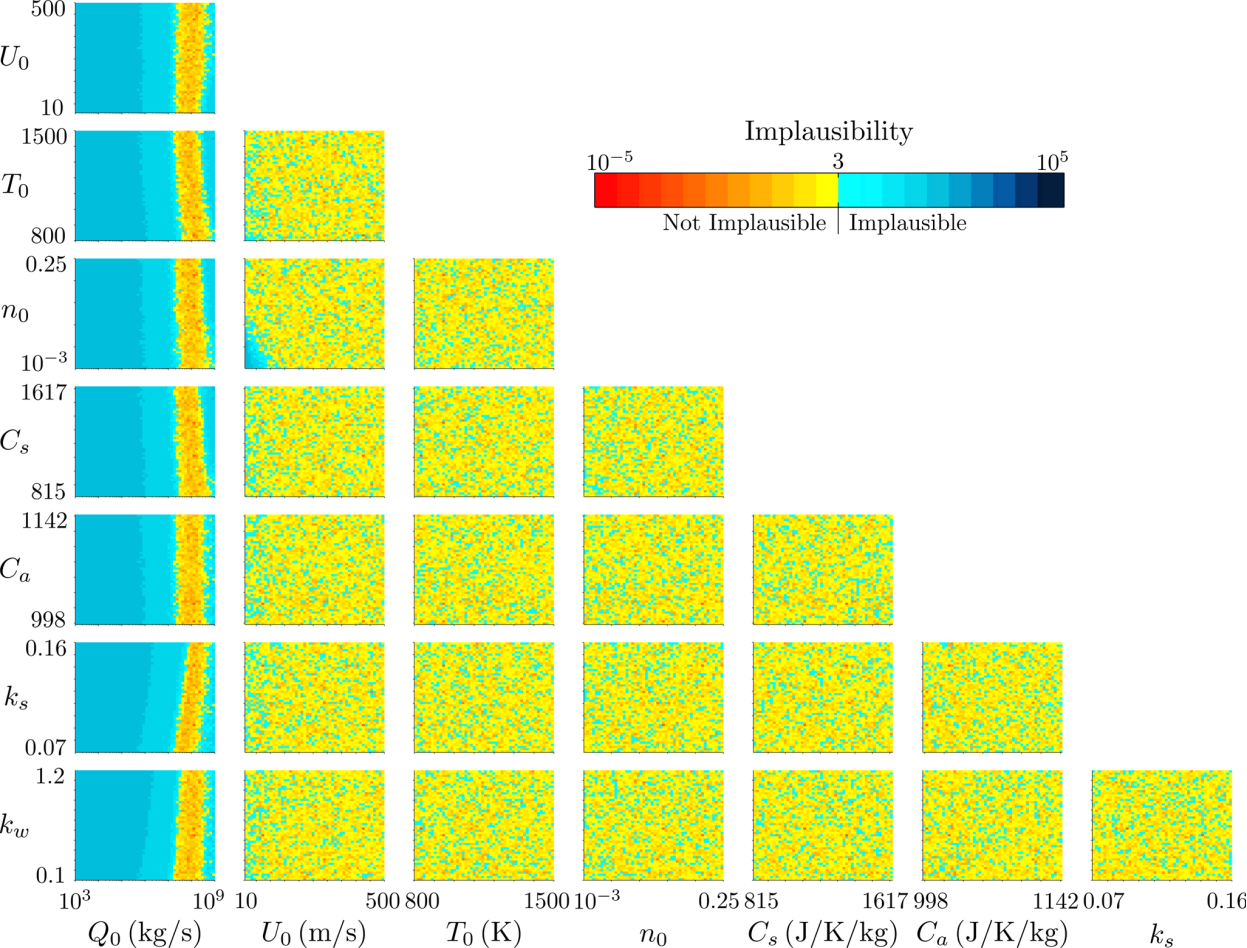
The projection of the minimum implausibility measure onto two-variable planes for history matching to the radar-derived plume height at Grímsvötn at 0500 on 22 May 2011

**Fig. 12:**
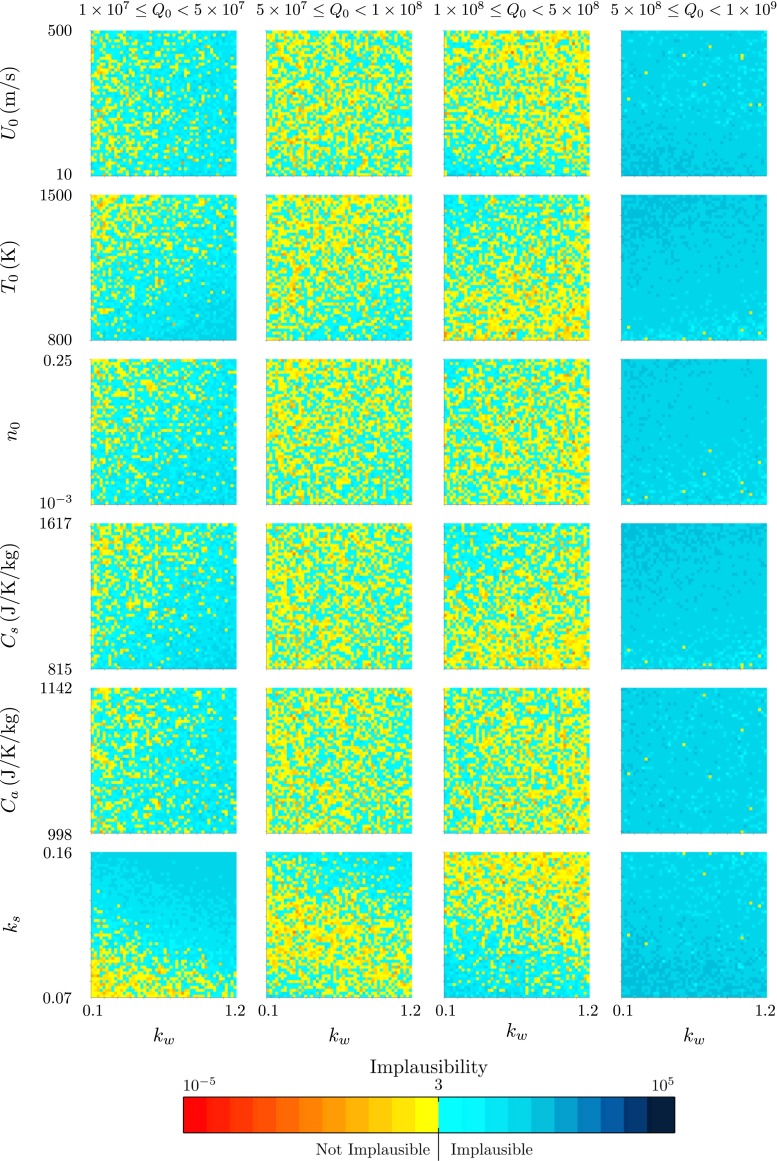
The projection of the minimum implausibility measure onto two-variable planes within intervals of the source mass flux *Q*_0_ for history matching to the radar-derived plume height at Grímsvötn at 0500 on 22 May 2011

The history-matching results for the Grímsvötn eruption at 0500 on 22 May 2011 are shown in Figs. 11 and 12. The range of not-implausible source mass flux ($1.6\times 10^{7} \leq Q_{0}^{\ast } \leq 8.5\times 10^{8}\,\mathrm {kg/s}$) occurs at markedly increased values in comparison to the results from the Eyjafjallajökull eruption (Figs. 5, 6 and 9). The dependence of the not-implausible source mass flux $Q_{0}^{\ast }$ on the wind entrainment coefficient *k*_*w*_ is much weaker for the Grímsvötn example than for the Eyjafjallajökull cases. We also see that the region of the input space with both *U*_0_ and *n*_0_ at the low end of their range of values that is ruled out by the history matching has increased in extent. Thus, low velocities at the vent are implausible unless the ejected material has an abundance of gas. Figure 12 shows a strong inverse relationship between the entrainment coefficients that result in plausible matches between the model prediction and the plume height observation; high values of the no-wind entrainment coefficient *k*_*s*_ are ruled out unless the wind entrainment coefficient *k*_*w*_ and source mass flux *Q*_0_ are sufficiently high.

## Discussion

**Fig. 13:**
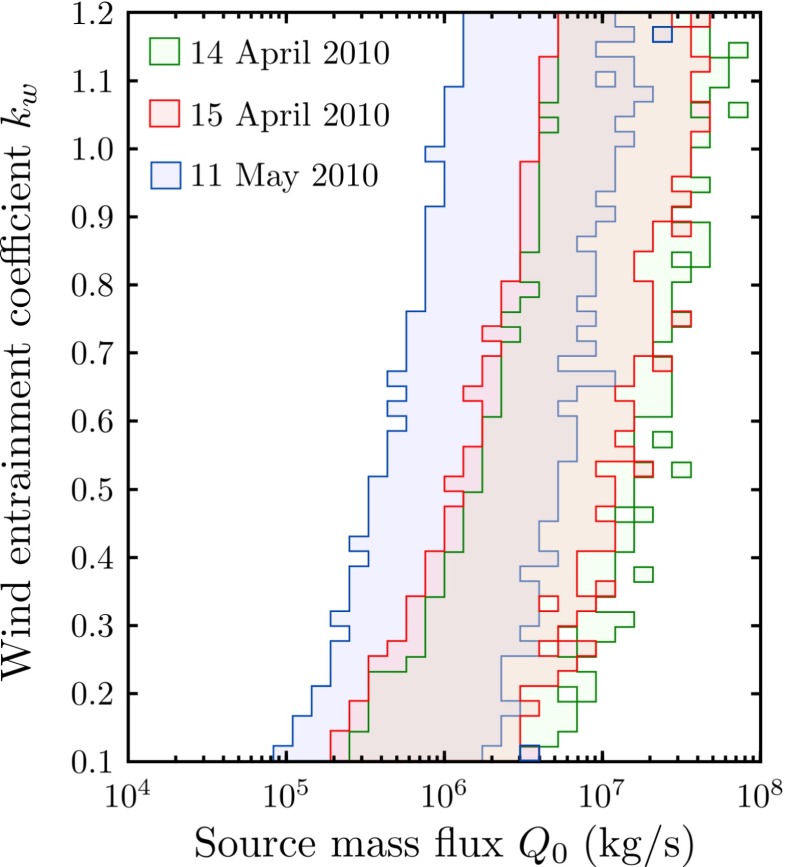
Not-ruled-out values of the source mass flux *Q*_0_ as a function of the wind entrainment coefficient *k*_*w*_ using a 3*σ*-threshold, for 14 April 2010 (*green*), 15 April 2010 (*red*) and 11 May 2010 (*blue*)

The observations of plume height from Eyjafjallajökull at 1200 on 14 April 2010 and 0000 on 15 April 2010 differ by approximately 3.3 km. Woodhouse et al. ( 2013) suggests that plume height differences on these occasions could be accounted for by changes in the meteorological conditions with the volcanic source conditions held constant. The history matching uncertainty analysis supports the hypothesis that the volcanic source conditions (particularly the source mass flux) did not change substantially between 1200 on 14 April and 0000 on 15 April (Fig. 13). The change in the atmospheric input to the model alone is sufficient to describe the difference in the plume heights. Importantly, this conclusion is independent of the value taken for the wind entrainment coefficient *k*_*w*_ (Fig. 13).

The large uncertainty in the wind entrainment coefficient *k*_*w*_ results in an inability to place tight constraints on the source mass flux *Q*_0_, and history matching allows us to quantify this uncertainty. The distribution of the source mass flux that is considered plausible following history matching for each of the Eyjafjallajökull cases spans two orders of magnitude (Fig. 13). Constraining the wind entrainment coefficient reduces the range of values for the source mass flux that is not ruled out of the input space. For example, taking 0.72≤*k*_*w*_≤0.9 as suggested from observations of chimney plumes (Hoult et al. 1969; Fay et al. 1970), the source mass flux on 14 April that is not-ruled-out by history matching spans the range 3.0×10^6^≤*Q*_0_≤4.4×10^7^ kg/s (Fig. 13). In contrast, taking the range 0.6≤*k*_*w*_≤0.71 as found from laboratory experiments on plumes in a uniform cross-wind (Hewett et al. 1971; Hoult and Weil 1972; Contini and Robins 2001), the range of source mass flux on 14 April is 2.3×10^6^≤*Q*_0_≤2.5×10^7^ kg/s (Fig. 13). Thus, when constraining the wind entrainment coefficient to empirically determined values, the range of plausible values for the source mass flux still covers an order of magnitude, due primarily to observational uncertainty in the radar plume heights. It is notable that applications of plume models have often used lower values of the wind entrainment coefficient, with Degruyter and Bonadonna ( 2012) and Mastin ( 2014) adopting a value *k*_*w*_=0.5, whereas Barsotti et al. ( 2008) and Barsotti and Neri ( 2008) use *k*_*w*_=0.6.

During the second explosive phase of the Eyjafjallajökull eruption, the eruption strength was typically deduced to be lower than during the first explosive phase (Gudmundsson et al. 2012) and the plume was strongly wind affected (Gudmundsson et al. 2012; Degruyter and Bonadonna 2012; Woodhouse and Behnke 2014). The plume on 11 May 2010 reached an altitude of 5 km, similar to the plume height observed on 15 April during the first explosive phase, but the wind speed on 11 May was lower than on 15 April (Fig. 4). Additionally, the moisture content of the atmosphere on these occasions differed. The history matching uncertainty analysis demonstrates that the different atmospheric conditions are sufficient to result in an order of magnitude difference in the prediction of the source mass flux between the first explosive phase (14 and 15 April 2010) and the plume on 11 May 2010 (Fig. 13).

**Fig. 14:**
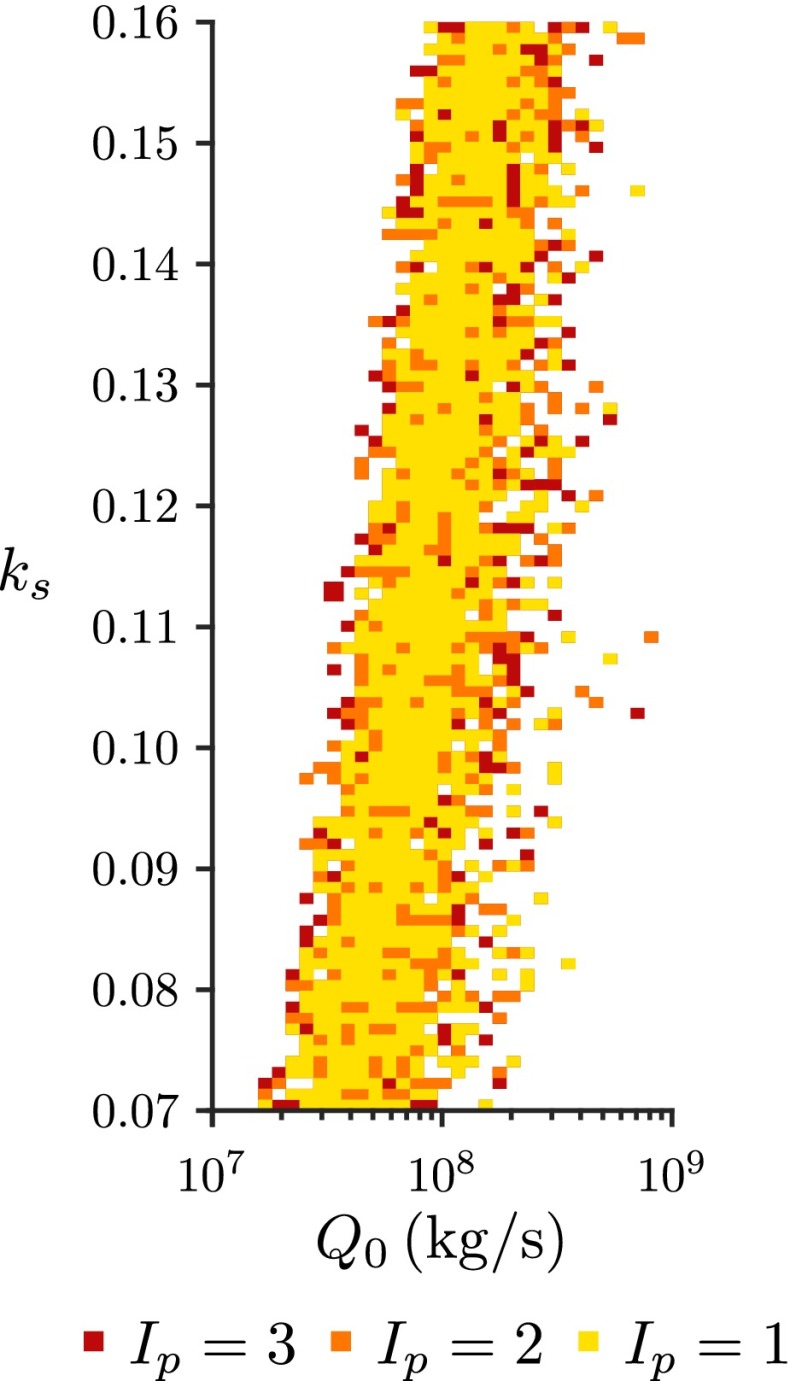
Not-ruled-out values of the source mass flux *Q*_0_ as a function of the no-wind entrainment coefficient *k*_*s*_ for three values of the implausibility threshold *I*_*p*_ following history matching to the height of the plume from Grímsvötn at 0500 on 22 May 2011. The threshold *I*_*p*_=2 is appropriate when $\text {Var}\left (\epsilon ^{obs}\right ) = 160\,\mathrm {m}^{2}$, but for the threshold *I*_*p*_=1 to be appropriate the model discrepancy must also be decreased to $\text {Var}\left (\epsilon ^{md}\right ) = 100\,\mathrm {m}^{2}$

For the high rising plume from the Grímsvötn eruption on 22 May 2011, the low wind speed and strength of the eruption resulted in a plume that was much less affected by wind than the plumes from the Eyjafjallajökull eruption. The history-matching results reflect this, with weak dependence of the source mass flux predictions on the wind entrainment coefficient *k*_*w*_ and a stronger dependence on the no-wind entrainment coefficient *k*_*s*_ than is found for the weak plumes at Eyjafjallajökull. The observational error for the Grímsvötn eruption is substantial, despite the use of a proximally deployed mobile radar, so the range of source mass flux that is not ruled out by the history matching remains large (1.6×10^7^≤*Q*_0_≤8.5×10^8^ kg/s). With improvements to the operational strategy of the mobile X-band radar in Iceland, it is anticipated that the observational error on plume heights will be greatly reduced during future eruptions. However, without improved characterization of the model parameters and model inputs, the reduction in the observational uncertainty will result in only a modest decrease in the range of the source mass flux that is not ruled out in history matching. For example, if the observational uncertainty could be reduced to Var(*𝜖* ^*obs*^) = 160 m ^2^ (so *α*=0.1), then the threshold on the implausibility measure is *I*_*p*_=2.0 from Eq. 5, but there is only a slight reduction in the range of plausible values of the source mass flux, as illustrated in Fig. 14. We note from Eq. 5 that reducing the observational uncertainty to zero (which cannot be achieved in practice) gives an implausibility threshold *I*_*p*_=1.86, and to further constrain the space of plausible model inputs there must be a reduction in the model discrepancy, through reduced uncertainty in the observations of the atmospheric conditions (the model forcing) and improvement in the model to reduce the implicit structural uncertainty.

In our analysis, we have not varied the meteorological input. The atmospheric forcing has a strong influence on model predictions (Woods 1993; Glaze and Baloga 1996; Bursik 2001; Degruyter and Bonadonna 2012; Woodhouse et al. 2013) and therefore uncertainty in the meteorological input can impact on the ability of the model to reproduce observations. Here, we have incorporated the uncertainty in the meteorological forcing through the model discrepancy in the implausibility measure. Further examination of the uncertainty in meteorological forcing can be achieved by coupling the plume model to an NWP model. The inputs to the NWP model can then be included in a history matching uncertainty analysis, but this will likely increase substantially the dimension of the space of model inputs.

Two algebraic expressions have been proposed to relate source conditions to the plume height for volcanic plumes rising in a wind-field (Degruyter and Bonadonna 2012; Woodhouse et al. 2013), which can be considered as surrogate models of the integral plume model. The numerical solution of the integral model is sufficiently rapid that the surrogate models offer little benefit in evaluation but introduce new uncertainties, e.g. parameter uncertainty from new parameters that are introduced, and structural uncertainty from the representation of atmospheric profiles by averaged values. However, the algebraic expressions provide some insight into the relationships revealed in the history matching.

The relationships proposed by Degruyter and Bonadonna ( 2012) and Woodhouse et al. ( 2013) each have the form,

6 $$\begin{array}{@{}rcl@{}} Q_{0} &=& \frac{2^{5/2}\pi {k_{s}^{2}}}{{z_{1}^{4}}}\\ &&\times\!\left[\!\!\frac{P_{A0}C_{a}}{g\left( \left( C_{v}n_{0}+C_{s}\left( 1-n_{0}\right)\right)T_{0}-C_{a}T_{A0}\right)R_{a}}\!\!\right]\!N^{3}\!H^{4} \!f(\mathcal{W}), \end{array} $$

where *P*_*A*0_ and *T*_*A*0_ denote the atmospheric pressure and temperature at the vent, respectively, *N* is a representative atmospheric buoyancy frequency, given for example by the average of the buoyancy frequency over the height of the plume (Degruyter and Bonadonna 2012) and *z*_1_ is a calibration parameter (Morton et al. 1956 give *z*_1_=2.8 from numerical solutions of an integral model of pure plumes, i.e. with boundary conditions corresponding to a point source of buoyancy with no flux of mass or momentum). Note, here we have inverted the expression presented in Woodhouse et al. ( 2013) to give the source mass flux as a function of the plume height, and we have explicitly included source thermodynamic properties in the prefactor of the scaling relationship (see Woodhouse et al. 2013). The effect of wind is described by the function $f(\mathcal {W})$ which is a monotonic increasing function of a dimensionless measure of the wind speed $\mathcal {W}$. The surrogate models differ in the specification of the dimensionless wind speed $\mathcal {W}$, and the form of the function *f*. Degruyter and Bonadonna ( 2012) propose

7 $$ f(\mathcal{W}) = 1 + \frac{{z_{1}^{4}}}{2^{5/2}}.\frac{{k_{w}^{2}}}{6{k_{s}^{2}}}\mathcal{W}, \quad \text{with} \quad \mathcal{W} = \frac{\bar{v}}{\bar{N}H},  $$

while Woodhouse et al. ( 2013) suggest

8 $$ f(\mathcal{W}) = \left( \frac{1 + b\mathcal{W} + c\mathcal{W}^{2}}{1+ a\mathcal{W}}\right)^{4}, \quad \text{with} \quad \mathcal{W} = 1.44\frac{\dot{\gamma}}{N}.  $$

Here, $\bar {v}$ and $\bar {N}$ are the wind speed and buoyancy frequency averaged over the plume height (Degruyter and Bonadonna 2012), $\dot {\gamma }$ is representative of the shear rate of the atmospheric wind and *a*=0.87+0.05*k*_*w*_/*k*_*s*_, *b*=1.09+0.32*k*_*w*_/*k*_*s*_ and *c*=0.06+0.03*k*_*w*_/*k*_*s*_ (Woodhouse et al. 2013). The functional form Eq. 7 is obtained by Degruyter and Bonadonna ( 2012) from a linear combination, with equal weights, of the plume rise height relationship of Morton et al. ( 1956) for plumes in a quiescent ambient (when $\mathcal {W}\equiv 0$) and the asymptotic expression of Hewett et al. ( 1971) for the rise height of a pure plume in a uniform cross wind that is appropriate for $\mathcal {W}\gg 1$ (Hewett et al. 1971). In contrast, the more complex functional form in Eq. 8 emerges as Woodhouse et al. ( 2013) fit their algebraic expression to numerical solutions of the integral plume model in a standard atmosphere over a range of values of $\mathcal {W}$ rather than employing an asymptotic relationship. However, the physics captured by the algebraic relationships of Degruyter and Bonadonna ( 2012) and Woodhouse et al. ( 2013) are essentially identical; the rise height of the plume decreases as the shear rate of the wind increases for a fixed source mass flux, due to enhanced mixing.

Some of the correlations between model inputs found in history matching can be anticipated from the algebraic relationships. Indeed, the uncertainty in plume height observations results in a range of heights *H* to be input into the expressions (7) and (8), and therefore a range of values for the source mass flux *Q*_0_ is expected. In a weak wind field, the wind parameter $\mathcal {W}$ is small and $f(\mathcal {W})\approx 1$, so the predicted source mass flux depends on the no-wind entrainment coefficient, *k*_*s*_. In contrast, in a strong wind field, $\mathcal {W}>0$, and a dependence of the source mass flux on the wind entrainment coefficient *k*_*w*_ can be anticipated through the function $f(\mathcal {W})$. A weak dependence of the source mass flux on the source temperature *T*_0_, and the source gas mass fraction *n*_0_, is expected from Eq. 6, but changes in these source conditions can be compensated by variation in the uncertain thermodynamic parameters, *C*_*s*_ and *C*_*a*_.

Our analysis identifies a region of input space with low exit velocity and low gas content that is considered implausible. The surrogate algebraic expressions cannot capture this region, as the relationships do not account for the momentum flux of the erupted material. However, the existence of such an implausible region can be explained by consideration of collapse criteria for wind blown plumes (Degruyter and Bonadonna 2013) which shows that, if the erupted material has insufficient kinetic energy relative to its potential energy at the vent, then the erupted material does not become buoyant.

### Implications for observations of volcanic plumes

The history matching performed in this study uses only a single datum to characterize the plume, i.e. the radar-derived plume height observation. It would be expected that incorporating additional observations would further constrain model inputs to produce better estimates of the source mass flux.

Acoustic measurements in the infrasonic frequency range have been used as volcano monitoring tools (see Johnson and Ripepe 2011 and Fee and Matoza 2013 for comprehensive reviews of volcano infrasound acoustics) and, through application of an acoustic source model, estimates of the ejection velocity (Caplan-Auerbach et al. 2010; Ripepe et al. 2013) or the ratio of the vent radius to the ejection velocity (Matoza et al. 2009; Fee and Matoza 2013) can be made. Observations of the exit velocity at the volcanic vent, including measurement uncertainty, could be incorporated into a history matching analysis. Our results, however, suggest that observational constraints on the exit velocity will not substantially reduce the size of the model input space that is not-ruled-out by history matching, with the exception of, perhaps, ruling out extremely low values of the gas mass fraction at the vent if the measured velocity is sufficiently low (Figs. 5, 6, 9 and 11). In contrast, measurements of the velocity at more than one point in the plume may be useful in ruling out more of the input space by, for example, indicating super-buoyancy (Woods 1988; Bjornsson et al. 2013).

In an analysis of the infrasound record during the second explosive phase (5–17 May 2010) of the Eyjafjallajökull eruption, Ripepe et al. ( 2013) adopts a dipole acoustic source model to determine the source mass flux from the measured acoustic pressure, assuming values of the vent radius (obtained from satellite images following the end of the eruption), volatile content of the magma, gas density (i.e. gas composition and temperature) and magma density (Ripepe et al. 2013). Ripepe et al. ( 2013) finds a source mass flux *Q*_0_ that varies in the range 5.7×10^5^– 1.2×10^6^ kg/s over the period 5–17 May 2010, and on 11 May an estimate of *Q*_0_≈1.1×10^6^ kg/s is obtained. This estimate is consistent with the not-ruled-out range of *Q*_0_ obtained from our history matching analysis (Fig. 13).

Photographs of plumes, either in the visible spectrum (Sparks and Wilson 1982; Formenti et al. 2003; Arason et al. 2011; Bjornsson et al. 2013) or using infrared (Patrick et al. 2007; Patrick 2007; Sahetapy-Engel and Harris 2009; Delle Donne and Ripepe 2012; Webb et al. 2014) or ultraviolet (Yamamoto et al. 2008) cameras, provide further observational data on plume dynamics, such as plume trajectory and growth. The growth of the plume is directly linked to the entrainment of atmospheric air (Morton et al. 1956; Turner 1986) and therefore observations can be used to estimate entrainment coefficients (Sparks and Wilson 1982; Patrick 2007). Images of the volcanic plumes from the Eyjafjallajökull eruption in 2010 (Arason et al. 2011; Bjornsson et al. 2013) and the Grímsvötn eruption in 2011 (Petersen et al. 2012) could be used within a history matching analysis in an attempt to further constrain the model inputs.

The history-matching analysis indicates a weak relationship between source temperature *T*_0_ and source mass flux *Q*_0_, suggesting that the incorporation of observations of temperature might further reduce the size of the not-ruled-out model input space. Thermal imaging of volcanic plumes using infrared (Patrick et al. 2007; Patrick 2007; Sahetapy-Engel and Harris 2009; Delle Donne and Ripepe 2012) and ultraviolet (Yamamoto et al. 2008) cameras have been used to examine the dynamics of Vulcanian and Strombolian eruptions and assess predictions of simple models of buoyant thermals and starting plumes. In addition to determining the temperature variation of the plume, thermal imaging can provide measurements of the velocity profile and the transition height from the gas-thrust region to buoyant motion (Patrick et al. 2007; Patrick 2007; Sahetapy-Engel and Harris 2009; Delle Donne and Ripepe 2012), and the ash concentration in dilute plumes (Yamamoto et al. 2008).

In addition to incorporating new observations of plume dynamics, it is also possible to include observations from other sources to constrain the model inputs by defining new implausibility measures. In particular, the source mass flux can be estimated through tephra sampling during an eruption (e.g. Bonadonna et al. 2011), measurements of ground deformation (e.g. Kozono et al. 2013) and satellite observations of the growth of umbrella clouds (e.g. Pouget et al. 2013). Each of these techniques rely on a model to extract the mass flux from direct measurements, and therefore history matching is a useful tool to assess the uncertainty in these estimates.

### Implications for plume modelling and volcanic source flux estimation

Volcanic ash transport and dispersion models (VATDMs) require an estimate of the source mass flux or total mass erupted in order to forecast the concentration of ash in the atmosphere (Folch 2012). Uncertainties in the description of the source terms are propagated through the VATDM (Scollo et al. 2008; Bonadonna et al. 2012; Bursik et al. 2012; Folch 2012; Denlinger et al. 2012). If appropriate observations are available, then inversion methods for data assimilation can be used to refine the estimates of the source term (e.g. Stohl et al. 2011; Bursik et al. 2012; Denlinger et al. 2012; Kristiansen et al. 2012; Webster et al. 2012). Characterizing and quantifying the uncertainty in the source mass flux is essential to producing robust forecasts of ash dispersion for hazard management (Bonadonna et al. 2012).

It is clear from the history-matching uncertainty analysis that, with our current limited understanding of entrainment into wind-blown volcanic plumes, estimates of the source mass flux are imprecise. In this first analysis, we have retained a large range of values for the entrainment coefficients (indeed, a larger range than found in existing experimental studies) to avoid discounting plausible values from the study, and have taken ranges for *k*_*s*_ and *k*_*w*_ and no further probability judgements to let the observations induce constraints. These choices could be relaxed in a subsequent analysis, curtailing the range of values or constructing probability distributions for the entrainment coefficients, to reflect our confidence in experimental calibrations of these model parameters. This expert judgment is very commonly used in inversion studies to the extent that several model inputs and parameters are set at fixed values.

Our results indicate that a reduced range for the wind entrainment coefficient *k*_*w*_ will result in a reduction in the range of values of the source mass flux that are considered not-implausible. However, we must exercise caution in constraining model inputs. Much can be learned from laboratory experiments, but the application of parameter values calibrated in a well-controlled laboratory setting to the complicated natural environment can be problematic. For example, experiments to calibrate the wind-entrainment coefficient *k*_*w*_ have used uniform cross-wind conditions (e.g. Hewett et al. 1971; Hoult and Weil 1972; Contini and Robins 2001). These conditions are not representative of volcanic plumes that ascend to high altitude and therefore experience variations in the wind speed. The use of the current experimentally calibrated values of *k*_*w*_ for the volcanic setting is therefore questionable. New experiments examining non-uniform profiles of wind speed would be extremely valuable.

Calibration of the wind entrainment coefficient *k*_*w*_ could also be achieved through comparison of model predictions to field observations. For industrial chimney plumes, this approach gives a different range of values for *k*_*w*_ than found from laboratory experiments (Hoult et al. 1969; Fay et al. 1970). However, the volcanic setting is complicated by additional physical processes, corresponding to new model inputs and parameters that must also be calibrated. Comparison studies have demonstrated the applicability of integral models to describe volcanic plumes using entrainment coefficients calibrated in laboratory experiments (e.g. Sparks and Wilson 1982; Woods 1998; Woodhouse and Behnke 2014) but have not fully considered uncertainty. Thus, other values for entrainment coefficients are likely to also produce acceptable model predictions if model inputs are adjusted appropriately. An alternative approach, building on our analysis here, is to perform a history-matching uncertainty analysis incorporating additional observations (for example, the plume trajectory, growth rate, temperature variation etc.) and examine the distribution of entrainment coefficients that are not ruled out as implausible.

## Conclusions

Uncertainty is intrinsic in observations of natural processes and mathematical models used to understand and interpret them. When inverse modelling studies fail to fully account for uncertainty the resulting model predictions are often poor due to over-fitting of model inputs. Quantification of uncertainty requires an investigation of observational data and an analysis of the sources of uncertainty within the model formulation. History matching allows the uncertainty that arises from measurement and model development to be included into an inversion calculation. We have applied history matching to perform an uncertainty analysis of an integral model of volcanic plumes using observations of plume height obtained from weather radar. Our focus has been on assessing the utility of plume models to determine the source mass flux by inverse modelling, although the analysis provides insight into the ability (or inability) of the plume model to predict other properties of volcanological interest.

The history matching analysis has demonstrated that large observational uncertainty and poorly constrained model parameters result in an inability to constrain many of the model inputs. However, the source mass flux can be constrained by plume height measurements, and the strong influence of entrainment coefficients on the source mass flux predictions is evident. Experimental calibration of the entrainment coefficients reduces the range of the source mass flux that is considered not implausible following history matching, but observational uncertainty in radar-derived plume heights is such that the source mass flux estimates span an order-of-magnitude. New experimental studies and further comparisons of model predictions to observations are required to better constrain the wind entrainment coefficient in order to achieve improved estimates of the source mass flux.

Our analysis has employed only single-point measurements. The little information contained in the radar data severely limits the inferences that can be drawn on the model inputs. Incorporating additional measurements may provide observational constraints that allow the size of the not-ruled-out model input space to be reduced. The history matching uncertainty analysis is easily extended to incorporate additional observations by defining new implausibility measures and examining the intersection of the not-ruled-out spaces for each measure. This approach does not add to the computational expense as the model evaluation is performed without direct use of the observations, so a single sample of model evaluations can be used to compute a set of implausibility measures. However, a sequential analysis with new sampling designs as new observations and corresponding implausibility measures are added can allow improved resolution of the not-ruled-out space of model inputs.
